# Considerations and Caveats when Applying Global Sensitivity Analysis Methods to Physiologically Based Pharmacokinetic Models

**DOI:** 10.1208/s12248-020-00480-x

**Published:** 2020-07-17

**Authors:** Dan Liu, Linzhong Li, Amin Rostami-Hodjegan, Frederic Y. Bois, Masoud Jamei

**Affiliations:** Simcyp Division, Certara UK Limited, Level 2-Acero, 1 Concourse Way, Sheffield, S1 2BJ UK

**Keywords:** Global sensitivity analysis, Morris method, Sobol method, extended Sobol method, physiologically based pharmacokinetic (PBPK) modelling

## Abstract

Three global sensitivity analysis (GSA) methods (Morris, Sobol and extended Sobol) are applied to a minimal physiologically based PK (mPBPK) model using three model drugs given orally, namely quinidine, alprazolam, and midazolam. We investigated how correlations among input parameters affect the determination of the key parameters influencing pharmacokinetic (PK) properties of general interest, i.e., the maximal plasma concentration (*C*_max_) time at which *C*_max_ is reached (*T*_max_), and area under plasma concentration (AUC). The influential parameters determined by the Morris and Sobol methods (suitable for independent model parameters) were compared to those determined by the extended Sobol method (which considers model parameter correlations). For the three drugs investigated, the Morris method was as informative as the Sobol method. The extended Sobol method identified different sets of influential parameters to Morris and Sobol. These methods overestimated the influence of volume of distribution at steady state (*V*_ss_) on AUC24h for quinidine and alprazolam. They also underestimated the effect of volume of liver (*V*_liver_) for all three drugs, the impact of enzyme intrinsic clearance of CYP2C9 and CYP2E1 for quinidine, and that of UGT1A4 abundance for midazolam. Our investigation showed that the interpretation of GSA results is not straightforward. Dismissing existing model parameter correlations, GSA methods such as Morris and Sobol can lead to biased determination of the key parameters for the selected outputs of interest. Decisions regarding parameters’ influence (or otherwise) should be made in light of available knowledge including the model assumptions, GSA method limitations, and inter-correlations between model parameters, particularly in complex models.

**Electronic supplementary material:**

The online version of this article (10.1208/s12248-020-00480-x) contains supplementary material, which is available to authorized users.

**Figure:**
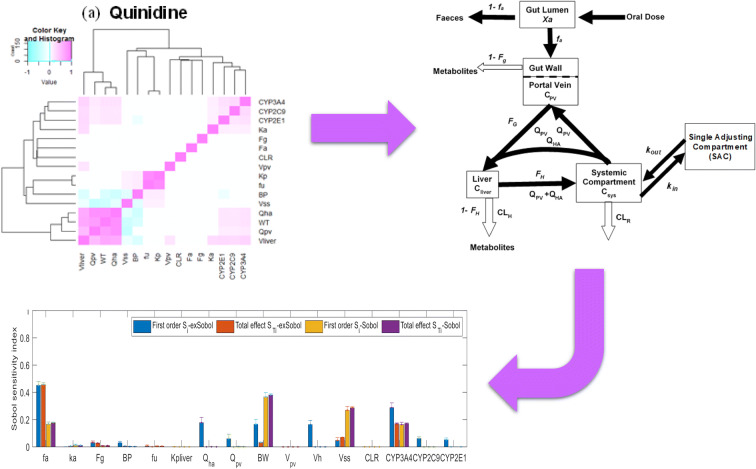
Graphical abstract

## Introduction

Sensitivity analysis, in its broad sense, has been widely used to identify and rank the most influential model parameters affecting the model outputs. Many factors determine the sensitivity of a model’s outputs to its parameters. Those are most notably: the number of input parameters, uncertainty, correlation, and interactions between them, and the non-linearity or non-monotonicity of the model (1). Correlation between two parameters means that the values of one parameter relate in some way to the values of the other, i.e., values of one parameter generally co-occur with certain values of the other. That implies that, for a given value of parameter *A* (correlated to parameter *B*) parameter *B* has a certain distribution, which in turn results in a given distribution of outcome *C*. If the value of *A* changes, so does the distributions of *B*, and the distribution of *C*. For example, Darwich et al. found that extremely high values of CYP3A intrinsic clearance can never occur simultaneously with high values of Michaelis-Menten constant (*K*_*m*_) of CYP3A and similarly no low values of CYP3A intrinsic clearance happens at the same time as having low *K*_*m*_ (2).

There are two types of sensitivity analyses: local analysis (LSA) and global analysis (GSA). Generally, local sensitivity analysis is based on model parameters set at baseline values with consideration of only minor perturbations to these baseline values within a specified range. LSA evaluates the model parameters’ impact on a specified output by altering one parameter at a time or a few simultaneously. It commonly does not consider parameters having a specific distribution and does not investigate the impact of parameters correlations on model outputs. GSA samples all parameters over an entire measurable parameter space (where the measure is a joint statistical distribution it can account for possible correlations), evaluating simultaneously the relative contributions of all model parameters and their potential interactions to a set of specified model outputs variance. Therefore, GSA is able to rank the importance of the model parameters while considering their uncertainty and correlations (3).

Various GSA methods have been proposed to support model design and parameter selection in the fields of engineering, biology, and clinical studies (4–16) some are listed here:

- Screening for elementary effects (Morris method) (7,17,18)
- Variance-based methods, such as Sobol’s method, Fourier amplitude sensitivity test (FAST), and extended FAST (eFAST) (7,11,17–19)
- Partial rank correlation coefficients method (PRCC) (11)
- Regional sensitivity analysis (RSA) (20,21)
- Dynamic identifiability analysis (DYNIA) (22)
- Density-based sensitivity analysis (PAWN) (21)
- Factors’ mapping and meta-modelling (17–19)

GSA methods can be further classified into two main categories: Restricted methods that assume independent input parameters, and methods that allow sampled parameters to be correlated. Most popular and commonly used GSA methods, e.g., Morris, PRCC, eFAST, and Sobol, assume the input parameters are non-correlated. These GSA methods provide information about the model structure or understanding the physiological mechanisms of biological responses. However, unrealistic parameter combinations, which bias sensitivity analysis, can be generated using random sampling with assumption of independent parameters. Furthermore, the validity of the sensitivity metrics or indices used by those methods depend on the assumption of non-correlated input parameters. If that assumption is not fulfilled, parameters determined as ‘influential’ may not be truly so. Fortunately, GSA methods accounting for correlated input parameters have recently been developed such as another extension of FAST (23,24), an extended high-dimensional model representation (HDMR) (3), or extended Sobol (9,25). Due to the complexity of these methods, they have had limited applications (26). A review of GSA algorithms is given in Supplemental Material. The main features of GSA algorithms assuming independent input parameters, and those accounting for correlation among parameters, are summarised in Table S1-2 and in the supplemental material, respectively.

In this work, we applied three GSA methods to a minimal physiologically based PK (mPBPK) model, in order to identify the most influential model parameters affecting three PK properties of three drugs dosed orally (quinidine, alprazolam, and midazolam). The three GSA methods were Morris screening, Sobol, and extended Sobol. The three PK properties studied were *C*_max_, the maximum plasma concentration, *T*_max_, the time at which *C*_max_ happens, and AUC, the area under the plasma concentration of the drugs. Quinidine and midazolam are Biopharmaceutical Classification System (BCS) class I, and alprazolam is a BCS class II drug. The model parameters identified as ‘important’ by the Morris and Sobol methods were compared to those determined by the extended Sobol method. This allowed us to assess the effect of ignoring parameters correlations when searching for influential parameters.

## Methods and Materials

### Morris Screening Method

Morris screening is simple to implement and does not require extensive computations. Implementation of Morris method is available in the supplemental material. Two metrics will be available from Morris method to assist parameter ranking, i.e, mean *μ* or μ* and standard deviation *σ*. A high *μ* or μ* indicates a parameter with an important overall influence on the model outputs; a high *σ* indicates either a parameter interacting with other factors or that its effect is non-linear. The magnitudes of *μ* and *σ* for each model parameter are relative to the others (12). Although they are not that informative, they still show some information about rate of changes.

### Sobol Method

The Sobol method is a variance-based type GSA method, which decompose the variance of the model outputs into sums of variances for combinations of input parameters of increasing dimensionality (27). Details of the derivation of Sobol sensitivity indices are explained in (28–32). Although there is no assumption about the relationship between the model inputs and outputs in variance-based GSA methods, they do assume that the input parameters are independent. Generally, when using Sobol, three sensitivity indices are calculated to determine the importance of input parameters:

- A first-order ‘main effect’ sensitivity index evaluating only the main influence of each parameter without considering the interaction with others
- A ‘total effect’ sensitivity index to assess the impact of each parameter including all possible interactions with others
- An ‘interaction’ index, which is the difference between total effect and main effect, representing only the contribution of parameters interactions

### Extended Sobol Method

The GSA method proposed by Kucherenko et al. (9) can consider models where the input parameters are correlated. The main (first-order) and total effect sensitivity indices, analogous to standard Sobol indices, are calculated using a copula-based method. Details of extended Sobol method can be found in (9). A brief description is also available in the Supplemental Material.

### Sensitivity Metrics to Detect Influential Parameters

For Morris screening, a global index (GI) ($\sqrt[2]{{\mu^{\ast}}^2+{\sigma}^2}$) was adopted to rank parameters (33). For the Sobol method, two sensitivity indices will be used for comparison, i.e., the first-order sensitivity index (*S*_*i*_) and the total sensitivity index (*S*_Ti_). Due to the difference between Sobol and extended Sobol in the presence of correlations of input parameters, the first-order sensitivity index (*S*_i,ext_) of estimated parameters using extended Sobol can be higher than the corresponding total effect sensitivity index (*S*_Ti,ext_) and can be bigger than 1, which mainly quantifies the partial variance contributed by uncorrelated variations (9). However, both indices can be used for ranking of the parameters.

For Sobol and the extended Sobol, only parameters with either first-order or total effect sensitivity index > 0.01 were considered as influential parameters and ranked, i.e., only parameters contribute > 1% to the total variance of outputs. Parameters with sensitivity index > 0.1, i.e., 10%, were considered as key parameters with significant impact on the model outputs. For Morris screening, input parameters were only ranked based on the GI metric, a relative comparison index, which does not tell what fraction of the total variance explained by the input parameters.

The performance of the Morris method depends on the number *l* of levels for each variable and on the number *r* of samples generated. Choices of *l* = 4 and *r* = 10 produce reasonable results (34). In this work, values of *l* = 10 and *r* = 1500 were adopted for a robust estimation. For Sobol, the number of random samples *N* higher than 1000 is recommended for a good estimation of the first-order and total effect Sobol indices (7). In this work, *N* = 8000 was used for the Sobol and the extended Sobol methods. Finally, 10 repetitions of Sobol and extended Sobol were performed to evaluate the variance of the calculated sensitivity indices. Thus, the total number of model evaluations was 80,000 using either Sobol or extended Sobol methods. Although, more samples or levels would potentially give more robust results, they would have required much more computational resource and calculation time.

### Minimal PBPK Model

**Fig. 1:**
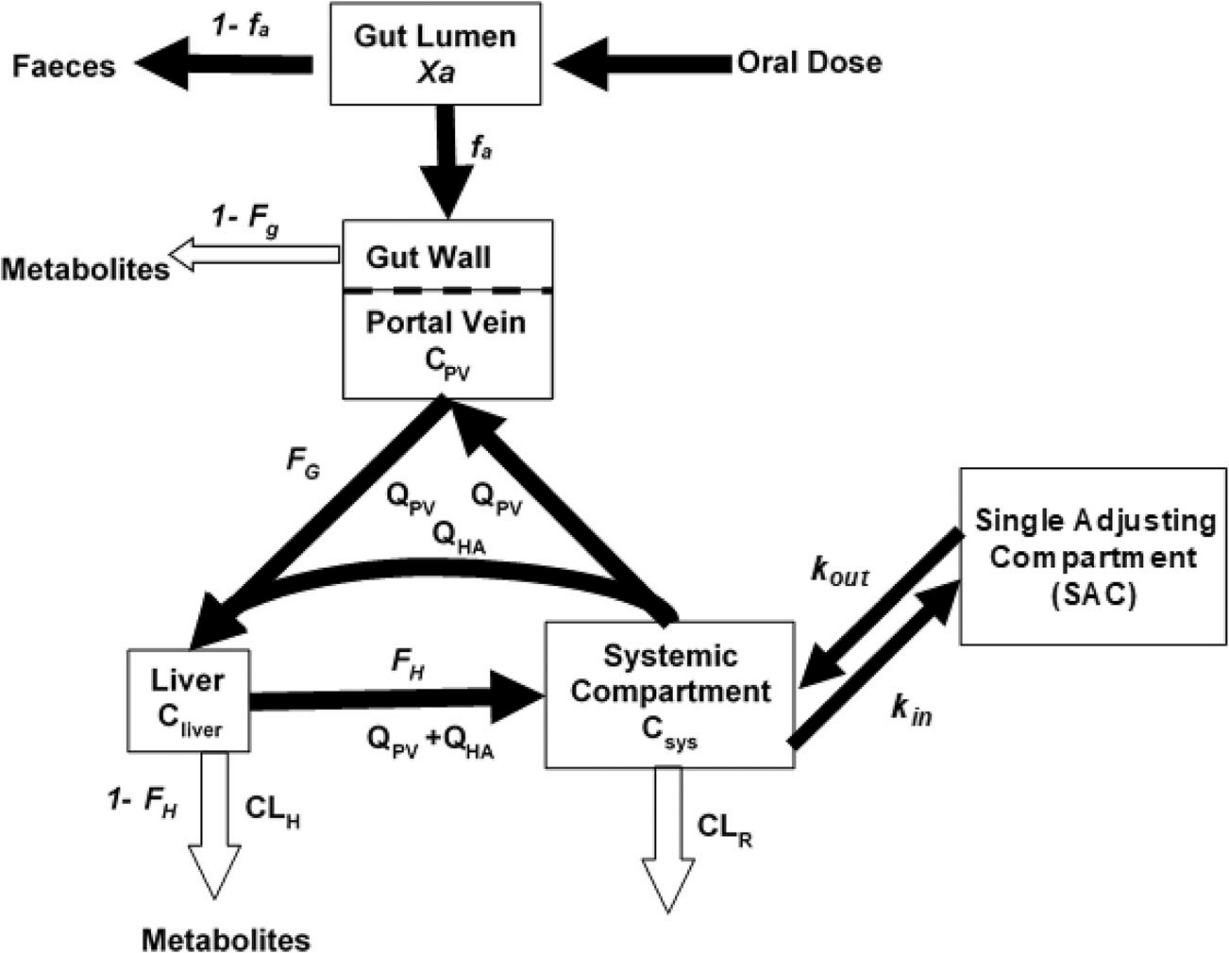
Illustration of the structure of an mPBPK model, please see the text for description of the parameters

A minimal PBPK model was used to simulate the PK properties of interest for orally administrated alprazolam, quinidine, and midazolam (see Fig. 1). Briefly, the mPBPK model equations are as follows (35):

1 $$ \frac{\mathrm{d}{\mathrm{C}}_{\mathrm{sys}}}{\mathrm{d}\mathrm{t}}=\frac{1}{V_{\mathrm{sys}}}\left[\left({Q}_{\mathrm{HA}}+{Q}_{\mathrm{p}\mathrm{v}}\right)\frac{C_{\mathrm{liver}}}{K{\mathrm{p}}_{\mathrm{liver}}/\mathrm{BP}}-\left({Q}_{\mathrm{HA}}+{Q}_{\mathrm{p}\mathrm{v}}\right){C}_{\mathrm{sys}}-\frac{\mathrm{C}{\mathrm{L}}_R{C}_{\mathrm{sys}}}{\mathrm{BP}}-{K}_{\mathrm{in}}{V}_{\mathrm{sys}}{C}_{\mathrm{sys}}+{K}_{\mathrm{out}}{V}_{\mathrm{sac}}{C}_{\mathrm{sac}}\right] $$

2 $$ \frac{\mathrm{d}{\mathrm{C}}_{\mathrm{sac}}}{\mathrm{d}\mathrm{t}}=\frac{1}{V_{\mathrm{sac}}}\left({K}_{\mathrm{in}}{V}_{\mathrm{sys}}{C}_{\mathrm{sys}}-{K}_{\mathrm{out}}{V}_{\mathrm{sac}}{C}_{\mathrm{sac}}\right) $$

3 $$ \frac{\mathrm{d}{\mathrm{C}}_{\mathrm{pv}}}{\mathrm{d}\mathrm{t}}=\frac{1}{V_{\mathrm{pv}}}\left({Q}_{\mathrm{pv}}{C}_{\mathrm{sys}}-{Q}_{\mathrm{pv}}{C}_{\mathrm{pv}}+{f}_a{k}_a{F}_g\mathrm{Dose}\times {e}^{-{k}_at}\right) $$

4 $$ \frac{\mathrm{d}{\mathrm{C}}_{\mathrm{liver}}}{\mathrm{d}\mathrm{t}}=\frac{1}{V_{\mathrm{liver}}}\left[{Q}_{\mathrm{p}\mathrm{v}}{C}_{\mathrm{p}\mathrm{v}}+{Q}_{\mathrm{HA}}{C}_{\mathrm{sys}}-\left({Q}_H+{Q}_{\mathrm{p}\mathrm{v}}\right)\frac{C_{\mathrm{liver}}}{\frac{K{\mathrm{p}}_{\mathrm{liver}}}{\mathrm{BP}}}-\mathrm{C}{\mathrm{L}}_{\mathrm{uintH}}{f}_u\frac{C_{\mathrm{liver}}}{K{\mathrm{p}}_{\mathrm{liver}}}\right] $$

5 $$ {V}_{\mathrm{ss}}=\frac{\left({K}_{\mathrm{pliver}}{V}_{\mathrm{liver}}+{V}_{\mathrm{sys}}\mathrm{BP}-{V}_{\mathrm{sac}}\mathrm{BW}\right)}{\mathrm{BW}} $$

6 $$ \mathrm{C}{\mathrm{L}}_{\mathrm{uintH}}={\Sigma}_{i=1}^M\mathrm{C}{\mathrm{L}}_{\mathrm{intCYP},i}+{\Sigma}_{i=1}^N\mathrm{C}{\mathrm{L}}_{\mathrm{intUGT},i} $$

where *C*_liver_ is the drug concentration in liver; *C*_pv_ is the blood drug concentration in portal vein; *C*_sys_ is the systemic blood concentration; *C*_sac_ is the drug concentration in the single adjusting compartment (SAC); *Q*_HA_ and *Q*_pv_ are the arterial and portal vein blood flow rates to the liver respectively; *V*_liver_, *V*_pv_, *V*_sys_, and *V*_sac_ represent volume of liver, portal vein, systemic compartment, and SAC (per kg of body weight) respectively; *K*_in_ and *K*_out_ are mass transfer rate constants in and out of SAC; *f*_*a*_ is the fraction of drug absorbed into enterocytes; *k*_*a*_ is the absorption rate constant; *F*_*g*_ is the fraction of drug escaping gut wall metabolism; *K*p_liver_ is the ratio of drug concentration in the liver to the plasma concentration (drug tissue partition coefficient in liver); BW is the body weight; BP is the blood to plasma ratio; *V*_ss_ is the volume of distribution at steady state (per kg of body weight); CL_u,inH_ is the unbound hepatic intrinsic clearance; CL_int, CYP_ and CL_int, UGT_ stand for drug intrinsic clearance contributed by CYP and UGT enzymes respectively; and CL_*R*_ is the renal clearance with respect to plasma. SAC was only used for modelling midazolam. *M* and *N* represent the number of contributing CYPs and UGTs for each drug metabolic clearance respectively.

Enzyme kinetics for each of the drugs were modelled in the mPBPK on the basis of their assigned elimination pathways in the Simcyp simulator (35). For quinidine three enzymes contribute to the hepatic intrinsic clearance CL_uintH_ = CL_int, CYP2E1_ + CL_int, CYP2C9_ + CL_int, CYP3A4_. For alprazolam, two enzymes contribute to the hepatic intrinsic clearance CL_uintH_ = CL_int, CYP3A4_ + CL_int, CYP3A5_. For midazolam, three enzymes contribute and CL_uintH_ = CL_int, CYP3A4_ + CL_int, CYP3A5_ + CL_int, UGT1A4_. In all cases, enzyme kinetics followed the Michaelis-Menten equation:

7 $$ {\mathrm{CL}}_{\operatorname{int},\mathrm{enz}}={A}_{\mathrm{enz}}\kern0.5em \times {\mathrm{V}}_{m,\mathrm{enz}}/\left({K}_{m,\mathrm{enz}}+{C}_{u,\mathrm{liver}}\right) $$

Here, *A*_enz_ is the abundance of enzyme (total pmol P450 or pmol UGT), *V*_*m*_ is the drug maximum metabolite formation rate constant (pmol/min/pmol of isoform for CYPs or UGT), *K*_*m*_ is Michaelis-Menten constant (μM), *C*_*u*,liver_ is the unbound drug concentration in liver (μM).

### Parameter Distributions and Ranges

The range of each model parameter is required by all three GSA methods, and distributions should also be specified for the Sobol and ext-Sobol methods. Furthermore, a parameter correlation matrix is required by the extended Sobol method (in the absence of any correlation this method’s results are similar to those of the Sobol method). In our case study, distribution and ranges of parameters were obtained from population simulations of quinidine, alprazolam, and midazolam pharmacokinetics, using the Simcyp simulator V16 default settings. Samples of parameters for 2000 healthy adult North European Caucasians (20–50 years old and 50% female) were generated and a normal, lognormal, or Weibull distribution was fitted to the generated data, for each parameter. The best-fitted distribution, on the basis of the lowest Akaike information criterion, was selected. As no variability for the volume of portal venous blood (*V*_pv_) and renal clearance (CL_*R*_) were initially considered a normal distribution with 10% coefficient of variation (CV) were assumed for each of these parameters. The same lower and upper limits of each parameter were set the same as the values used in the Simcyp simulator. The parameter distributions and ranges used for GSA are summarised in Tables I, II, and III for each drug, respectively.

**Table I:** The parameter values and distribution for quinidine

Parameters	Abbreviation	Unit	Values/distribution	Min	Max
Dose		Mg	200		
Fraction of absorption	*f*_*a*_	n/a	Weibull (8.86, 0.94)	1e-6	1
Absorption rate	*k*_*a*_	1/h	Lognorm (1.05, 0.09)	1e-6	10
Gut availability	*F*_*g*_	n/a	Weibull (46.3, 0.96)	1e-6	1
Blood to plasma concentration ratio	BP	n/a	Norm (0.89, 9.64e-5)	0.55	100
Fraction of unbound drug in plasma	*f*_*u*_	n/a	Lognorm (− 1.61, 7e-3)	1e-6	1
Liver tissue to plasma partition coefficient	*K*_pliver_	n/a	Norm (4.37, 3.95e-2)	1e-6	10
Hepatic CYP2E1 intrinsic clearance	CL_int,CYP2E1_	L/h	Lognorm (0.47, 4.23e-1)	1e-6	100
Hepatic CYP2C9 intrinsic clearance	CL_int,CYP2C9_	L/h	Lognorm (0.18, 4.27e-1)	1e-6	100
Hepatic CYP3A4 intrinsic clearance	CL_int,CYP3A4_	L/h	Lognorm (4.35, 2.59e-1)	1e-6	1000
Hepatic arterial blood flow	*Q*_HA_	L/h	Lognorm (3.05, 1.44e-2)	1e-6	50
Portal vein blood flow	*Q*_PV_	L/h	Lognorm (4.19, 1.05e-2)	1e-6	150
Body weight	BW	Kg	Lognorm (4.30, 3.8e-2)	30	200
Volume of portal vein	*V_pv_	L	Norm (0.008, 6.4e-7)	1e-6	0.15
Volume of liver	*V*_liver_	L	Lognorm (0.39, 2.97e-2)	0.1	5
Distribution volume in plasma	*V*ss	L/kg	Lognorm (0.63, 2.83e-2)	1e-6	5
Renal clearance with respect to plasma	*CL_*R*_	L/h	Norm (1.95, 3.8e-2)	1e-6	5

*Parameter was assumed to be normally distributed with 10% CV

**Table II:** The parameter values and distributions for alprazolam

Parameters	Abbreviation	Unit	Values/distribution	Min	Max
Dose		mg	0.5		
Fraction of absorption	*f*_*a*_	n/a	Weibull (8.86, 0.94)	1e-6	1
Absorption rate	*k*_*a*_	1/h	Lognorm (1.21, 0.09)	1e-6	10
Gut availability	*F*_*g*_	n/a	Weibull (512.33, 1)	1e-6	1
Blood to plasma concentration ratio	BP	n/a	Norm (0.84, 2.05e-4)	0.55	100
Fraction of unbound drug in plasma	*f*_*u*_	n/a	Lognorm (− 1.25, 5.4e-3)	1e-6	1
Liver tissue to plasma partition coefficient	*K*_pliver_	n/a	Lognorm (− 0.146, 4.9e-3)	1e-6	10
Hepatic CYP3A4 intrinsic clearance	CL_int,CYP3A4_	L/h	Lognorm (2.10, 0.26)	1e-6	100
Hepatic CYP3A5 intrinsic clearance	CL_int,CYP3A5_	L/h	Lognorm (1.58, 0.18)	1e-6	100
Hepatic arterial blood flow	*Q*_HA_	L/h	Lognorm (3.05, 1.44e-2)	1e-6	50
Portal vein blood flow	*Q*_PV_	L/h	Lognorm (4.19, 1.05e-2)	1e-6	150
Body weight	BW	kg	Lognorm (4.30, 3.8e-2)	30	200
Volume of portal vein	*V_pv_	L	Norm (0.008, 6.4e-7)	1e-6	0.15
Volume of liver	*V*_liver_	L	Lognorm (0.39, 2.97e-2)	0.1	5
Distribution volume in plasma	*V*ss	L/kg	Norm (0.76, 1.06e-2)	1e-6	5
Renal clearance with respect to plasma	*CL_*R*_	L/h	Norm (0.68, 4.6e-3)	1e-6	5

*Parameter was assumed to be normally distributed with 10% CV

**Table III:** The parameter values and distributions for midazolam

Parameters	Abbreviation	Unit	Values/distribution	Min	Max
Dose		mg	5		
Fraction of absorption	*f*_*a*_	n/a	Weibull (8.86, 0.94)	1e-6	1
Absorption rate	*k*_*a*_	1/h	Lognorm (1.05, 0.09)	1e-6	10
Gut availability	*Fg*	n/a	Norm (0.47, 0.01)	1e-6	1
Blood to plasma concentration ratio	BP	n/a	Norm (0.64, 1.05e-3)	0.55	100
Fraction of unbound drug in plasma	*f*_*u*_	n/a	Lognorm (− 3.46, 1e-3)	1e-6	1
Liver tissue to plasma partition coefficient	*K*_pliver_	n/a	Lognorm (− 0.21, 9.6e-3)	1e-6	10
Hepatic abundance of CYP3A4	*A*_CYP3A4_	pmol P450	Lognorm (15.84, 0.26)	1e6	1e8
Hepatic abundance of CYP3A5	*A*_CYP3A5_	pmol P450	Lognorm (15.72, 0.18)	1e6	1e8
Hepatic abundance of UGT1A4	*A*_UGT1A4_	pmol UGT	Lognorm (14.92, 0.18)	1e5	1e8
Maximum metabolite formation rate by CYP3A4 (1-OH pathway)	*V*_*m,*CYP3A4_	pmol/min/pmol of isoform	5.23^+^		
Maximum metabolite formation rate by CYP3A5 (1-OH pathway)	*V*_*m,*CYP3A5_	pmol/min/pmol of isoform	19.7^+^		
Maximum metabolite formation rate by CYP3A4 (4-OH pathway)	*V*_*m,*CYP3A4_	pmol/min/pmol of isoform	5.2^+^		
Maximum metabolite formation rate by CYP3A5 (4-OH pathway)	*V*_*m,*CYP3A5_	pmol/min/pmol of isoform	4.03^+^		
Maximum metabolite formation rate by UGT1A4	*V*_*m,*UGT1A4_	pmol/min/mg microsomal protein	445^+^		
Michaelis-Menten constant for CYP3A4 (1-OH pathway)	*K*_*m,*CYP3A4_	μM	2.16^+^		
Michaelis-Menten constant for CYP3A5 (1-OH pathway)	*K*_*m,*CYP3A5_	μM	4.16^+^		
Michaelis-Menten constant for CYP3A4 (4-OH pathway)	*K*_*m,*CYP3A4_	μM	31.8^+^		
Michaelis-Menten constant for CYP3A5 (4-OH pathway)	*K*_*m,CYP3A5*_	μM	34.8^+^		
Michaelis-Menten constant for UGT1A4	*K*_*m*,UGT1A4_	μM	40.3^+^		
Hepatic arterial blood flow	*Q*_HA_	L/h	Lognorm (3.05, 1.44e-2)	1e-6	50
Portal vein blood flow	*Q*_PV_	L/h	Lognorm (4.19, 1.05e-2)	1e-6	150
Body weight	BW	kg	Lognorm (4.30, 3.8e-2)	30	200
Volume of portal vein	*V_pv_	L	Norm (0.008, 6.4e-7)	1e-6	0.15
Volume of liver	*V*_liver_	L	Lognorm (0.39, 2.97e-2)	0.1	5
Distribution volume in plasma	*V*_*ss*_	L/kg	Norm (0.91, 4.09e-2)	1e-6	5
Renal clearance with respect to plasma	*CL_*R*_	L/h	Norm (0.085, 4.6e-3)	1e-6	5
Absorption rate constant for SAC	*K*_*i*n_	1/h	0.2^+^		
Eliminate rate constant for SAC	*K*_out_	1/h	0.25^+^		
Volume of distribution for SAC	*V*_sac_	L/kg	0.23^+^		

*Parameter was assumed to be normally distributed with 10% CV

^+^Values were fixed

Many of anatomical and physiological parameters, such as age, sex, body weight, volume of organ, enzyme abundance, and renal function, are inter-correlated. Prior knowledge on these correlations can be obtained through mechanistic understanding, in vitro or in vivo studies. They may be generated by obvious physiological processes or can be hidden in observed data in which case further investigations are needed to reveal such relationships. To simulate correlated parameter values, we used standard multivariate normal sampling, with a correlation matrix capturing the strength of the underlying links. That correlation matrix was estimated directly from the data set generated using the Simcyp simulator, which accounts for many known correlations among anatomical and physiological parameters. The correlation matrix, as a prior estimate of correlations among input parameters, was further adjusted by setting non-significant (*p* > 0.05) and very week (*r* < 0.1) correlation coefficients to zero.

## Results

### Evaluation of the Implementation of GSA Methods

The GSA methods and the mPBPK model were both coded in Matlab 2016b. The correctness of the Matlab implementations of these three GSA methods was validated using four test functions comparing the calculated sensitivity indices with the corresponding analytical solutions or published results. For the Sobol method, sensitivity indices were cross-checked using two test functions, i.e., the non-linear and non-monotonic Ishigami-Homma function and the g-function (Figure S1-3 in Supplemental material). The performance of the Morris method was also evaluated comparing the determined parameters importance with ranking of the analytical solutions (Figure S1-3 in Supplemental material). For the extended Sobol method, the calculated sensitivity indices were compared against the analytical solutions of a linear function and the published sensitivity indices of Ishigami-Homma function by Kucherenko et al. (9) (Figure S4-5 in Supplemental material). Results are summarised in the Supplemental material, and show good agreement between the calculated sensitivity indices, the analytical solutions, and the published results.

### Quinidine

**Fig. 2:**
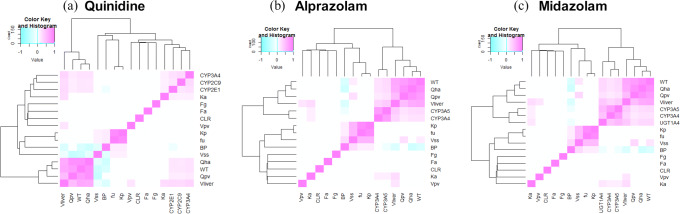
Correlation of model parameters based on the Simcyp simulator simulation results; **a** quinidine, **b** alprazolam, and **c** midazolam, please see the text for description of the parameters

**Fig. 3:**
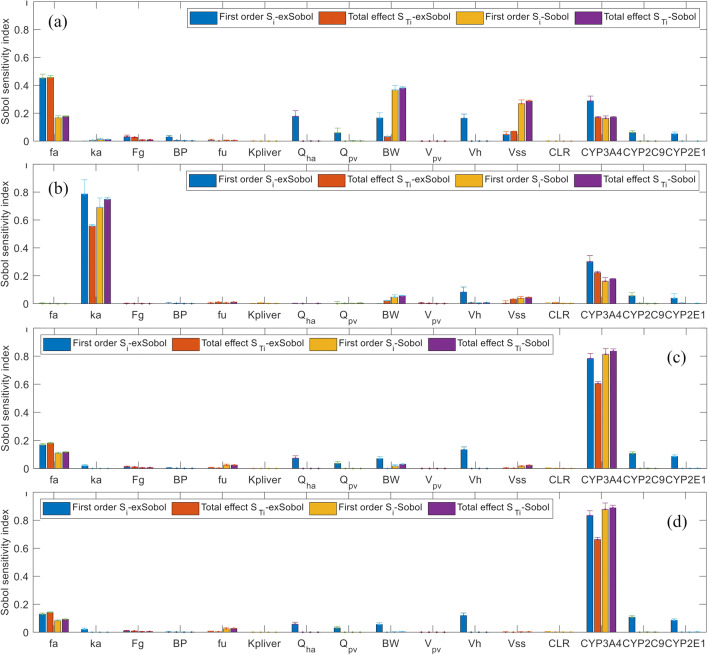
The extended Sobol indices vs. Sobol indices for quinidine: **a** *C*_max_, **b** *T*_max_, **c** AUC_24h_, and **d** AUC_48h_

In the mPBPK model of quinidine, inter-enzymes correlations was considered as well as the correlations between system parameters and enzyme intrinsic clearance (Fig. 2), the extended Sobol sensitivity indices (Table S3 in the supplement material, Fig. 3) suggest that:

1. *f*_*a*_, CL_int,CYP3A4_, *Q*_HA_, BW, and *V*_liver_, are the most important parameters affecting *C*_max_. CL_int,CYP2C9_, CL_int,CYP2E1_, *Q*_pv_, *V*_ss_, *F*_*g*_, and BP also have influence on *C*_max_.
2. *k*_*a*_, CL_int,CYP3A4_, and *V*_liver_ are the key influential parameters affecting *T*_max_. Second tier influential parameters, such as BW, CL_int,CYP2C9_, CL_int,CYP2E1_, *Q*_pv_, *V*_ss_, F_g_, and BP, can also result in notable changes of *T*_max_.
3. CL_int,CYP3A4_, *f*_*a*_, *V*_liver_, and CL_int,CYP2C9_ have significant impact on AUC_24h_ and AUC_48h_. Meanwhile, CL_int,CYP2E1_, *Q*_HA_, BW, *Q*_pv_, *k*_*a*_, and *F*_*g*_ also contribute to up to 10% variation of AUC each.

The half-life of quinidine is about 6–8 h. In 24 h around 87~93% of quinidine will be cleared from the body, suggesting AUC_24h_ ≈ AUC_48h_ ≈ AUC_∞_.

Not accounting for input parameters correlations, Morris and Sobol methods mostly agree and suggest that:

1. BW, *V*_ss_, *f*_*a*_, and CL_int, CYP3A4_ are the parameters significantly affecting *C*_max_.
2. *k*_*a*_ and CL_int, CYP3A4_ are the key influential parameters for *T*_max_. Extra influential parameters were BW, *V*_ss_, and *f*_*u*_.
3. CL_int, CYP3A4_, and *f*_*a*_ have most significant impact on AUC_24h_; additional parameters, such as BW, *f*_*u*_, and *V*_ss_, still contribute to variance of AUC_24h_. However, when considering AUC_48h_, the impact of *f*_*u*_ and *V*_ss_ diminished (Fig. 3, Table S3 and Figure S6 in supplemental material), as expected.

### Alprazolam

**Fig. 4:**
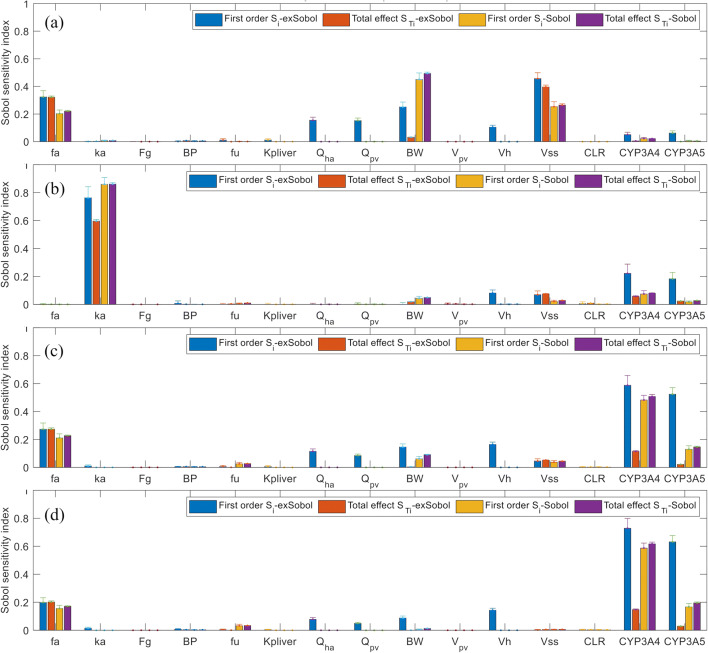
The extended Sobol indices vs. Sobol indices for alprazolam: **a** *C*_max_, **b** *T*_max_, **c** AUC_24h_, and **d** AUC_48h_

For alprazolam, the extended Sobol sensitivity indices (Table S4 in the supplement material, Fig. 4) indicate that:

1. *V*_ss_, *f*_*a*_, BW, *Q*_HA_, *Q*_pv_, and *V*_liver_ are the most important parameters affecting *C*_max_, followed by CL_int,CYP3A4_, CL_int,CYP3A5_, *f*_*u*_, and *K*p_liver_.
2. For *T*_max_, *k*_*a*_, CL_int,CYP3A4_, and CL_int,CYP3A5_ are the most important parameters, followed by *V*_liver_, *V*_ss_, and BW.
3. CL_int,CYP3A4_, CL_int,CYP3A5_, *f*_*a*_, *V*_liver_, BW, and *Q*_HA_ have significant impact on AUC_24h_. Other less influential parameters identified are *Q*_pv_, *V*_ss_, and *k*_*a*_.
4. Apart from *V*_ss_, the effect of which on AUC_48h_ became negligible, nearly the same sets of influential parameters have been identified for AUC_48h_ as those for AUC_24h_. Considering the half-life of alprazolam ~ 11.2 h, for a daily repeated oral dose only ~ 77% of the drug will be cleared from body in 24 h. However, about 95% of the drug will be cleared at 48 h, suggesting AUC_24h_ < AUC_48h_ ≈ AUC_∞_.

Likewise, Morris and Sobol methods (Fig. 4, Table S4 and Figure S7 in supplemental material) still agree and determined that:

1. BW, *V*_ss_, and *f*_*a*_ are the most important parameters for *C*_max_ followed by CL_int,CYP3A4_, which only has limited impact about < 10% variance.
2. *k*_*a*_ has been identified as the most influential parameters for *T*_max_. CL_int,CYP3A4_, BW, *V*_ss_, CL_int,CYP3A5_, and *f*_*u*_, also contribute to the variance of *T*_max_.
3. For AUC_24h_, CL_int,CYP3A4_, *f*_*a*_, and CL_int,CYP3A5_ are the most important parameters followed by BW, *V*_ss_, and *f*_*u*_.

Except *V*_ss_, the same sets of influential parameters were identified between AUC_24h_ and AUC_48h_. However, their relative importance changes (Table S4 in the supplement material).

### Midazolam

**Fig. 5:**
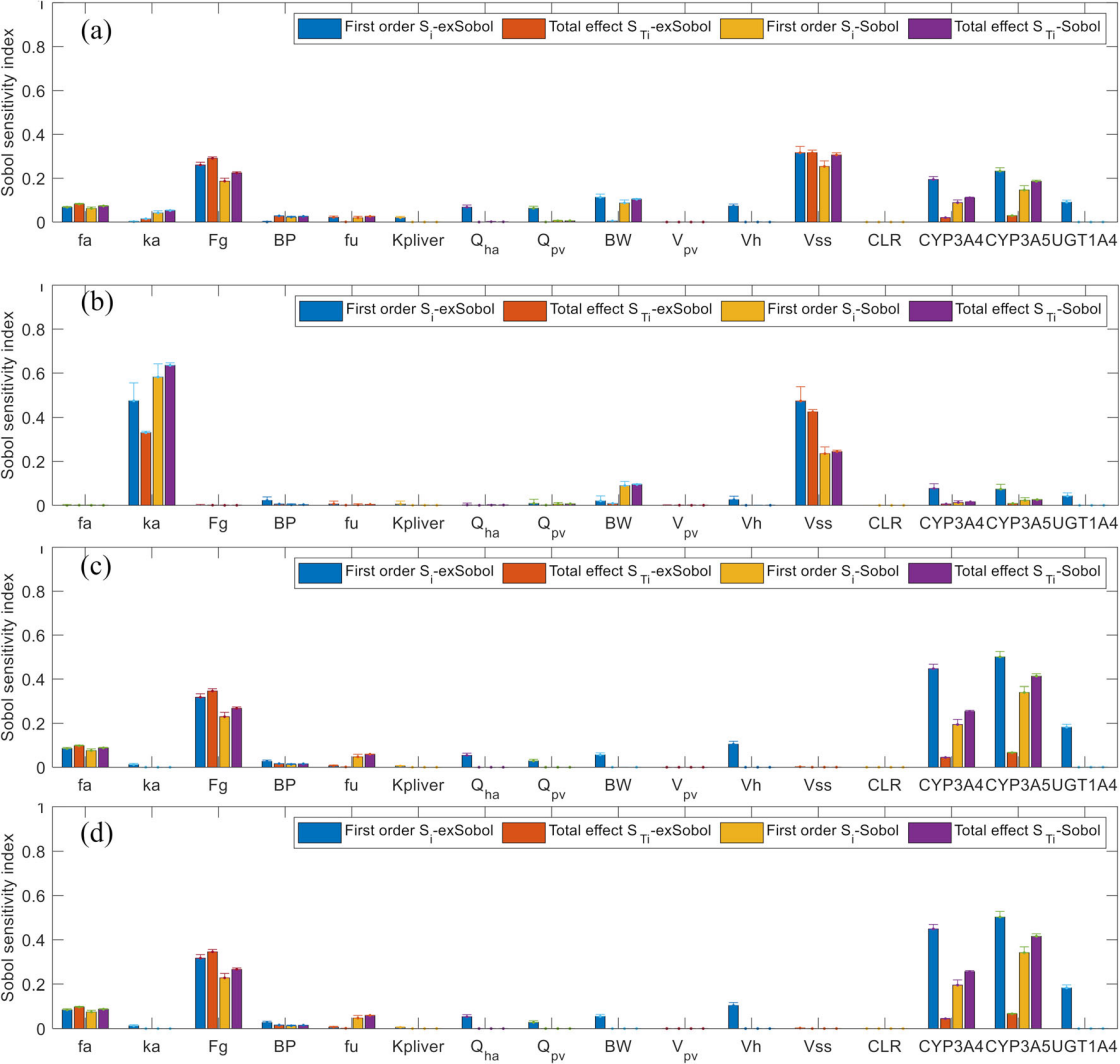
The extended Sobol indices vs. Sobol indices for midazolam: **a** *C*_max_, **b** *T*_max_, **c** AUC_24h_, and **d** AUC_48h_

For midazolam, extended Sobol sensitivity indices (Table S5 in the supplement material, Fig. 5) indicate that:

1. Apart from *V*_pv_ and CL_*R*_, all other model parameters will contribute to the variance of *C*_max_, in which *V*_ss_, *F*_*g*_, enzyme abundance A_CYP3A4_ and A_CYP3A5_, and BW, are the most important parameters.
2. *k*_*a*_ and *V*_ss_ are identified as the most significant parameters affecting *T*_max_, followed by *A*_CYP3A4_, *A*_CYP3A5_, *A*_UGT1A4_, *V*_liver_, BP, and BW.
3. *A*_CYP3A5_, *A*_CYP3A4_, *F*_*g*_, *V*_liver_, and *f*_*a*_ have significant impact on AUC_24h_. Other less effective parameters identified are BW, *Q*_HA_, *Q*_pv_, BP, and *k*_*a*_.
4. The same sets of influential parameters as for AUC_24h_ were recognised for AUC_48h_.

Dissimilar to quinidine and alprazolam, the key influential parameters (accounting for > 10% variance) for *C*_max_, *T*_max_, and AUC identified by Morris and Sobol are nearly the same as the extended Sobol (Fig. 5, Table S5 and Figure S8 in supplemental material). This may be due to the short terminal half-life of midazolam ~ 1.5 to 2.5 h. When giving a q.d. oral dose, ~ 99.9% midazolam will be eliminated in 24 h; hence, AUC_24h_ ≈ AUC_48h_ ≈ AUC_∞_.

## Discussion

Sensitivity analysis can help in narrowing down the number of parameters to be estimated prior to model calibration, avoiding model over-parameterisation, and assisting in model understanding or experimental design. In this study, Morris, Sobol, and extended Sobol methods were used to identify the most influential parameters of mPBPK models of quinidine, alprazolam, and midazolam affecting *C*_max_, *T*_max_, and AUC. We investigated the ability of the three methods to identify the contributions of all model parameters and their potential interactions to a set of specified model outputs, contributions coming not only from inter-individual variability but also from parameter correlations and model structure. Of the three drugs selected, (1) quinidine is an antiarrhythmic agent (36); (2) alprazolam one of the most commonly used drugs for short-term management of anxiety disorders with a relativley low clearance (37); (3) midazolam a widely used drug in anaesthesia (38,39) or as a preanesthetic medication (40). Although alprazolam and midazolam are both BCS class I drugs and cleared by the similar enzymes, their pharmacokinetics in the body are different, due to different clearance and volume of distribution (*V*_ss_).

GSA methods that are not considering input parameters’ correlation have been applied to PBPK modelling (6,12,15,16,41,42) or systems biology or pharmacology (1,13,19). For example, Fenneteau et al. applied PRCC to a PBPK model in order to identify important parameters affecting drug distribution in tissues with P-glycoprotein expressing aiming to reduce the uncertainty of model predictions (6). PRCC is robust for non-linear problem, but assumes monotonic relationships between parameter and outputs. If that assumption is not fulfilled, it is assessment can be inaccurate (11). McNally et al. proposed a two-stage GSA workflow for PBPK; in the first stage, the Morris method is applied to screen and select a subset of model input parameters, then eFAST is run to pinned down the most significant parameters (12). Similarly, Scherholz et al. proposed a two-stage global sensitivity analysis of the GastroPlus compartmental model, using Morris first and Sobol after that (15). Melillo et al. applied the Sobol method to identify key parameters influencing fraction absorbed and bioavailability for BCS class I–IV drugs (42). Variance-based method, e.g., Sobol and eFAST, do not assume model linearity or monotonicity and can evaluate interactions among input parameters. However, the fact that they do not consider correlations among input parameters is a major weakness of those GSA methods. To assess their performance, we have also applied the extended Sobol method, a GSA method considering parameter correlations. Our results support the assertion that GSA methods which do not take into account parameters correlations, when those in fact exist, can lead to wrong determinations of influential parameters. A comparison of the sensitivity indices given by the Sobol and extended Sobol methods with the analytical solution of a linear test function (Figure S4 in Supplemental material) suggests that Sobol, developed for models with non-correlated input parameters, will incorrectly determine parameter importance in the presence of moderate correlations among input parameters. Extended Sobol can properly recover the true main and total effects, assuming the parameters correlation is known and incorporated (test functions 3 and 4, and Figure S4 and S5 in supplemental material), hence correctly determining parameter contributions to the specified outputs.

As shown in Fig. 2, inter-enzymes correlations were considered in this work, as well as the correlations between system parameters and enzyme intrinsic clearance or enzyme abundance. However, the potential correlation between liver and gut enzymes were ignored, and *F*_*g*_ was considered as a single input parameter and independent of liver enzyme intrinsic clearance. *K*p_liver_ distribution was predicted using the Rodgers and Rowland method in the Simcyp simulator, indicating that there is a strong correlation between *K*p_liver_ and *f*_*u*_ with coefficient of determination *R*^2^ ~ 1. In order to individually explore the impact of *f*_*u*_ and *K*p_liver_ on *C*_max_, *T*_max_, and AUC, a strong correlation of 0.9 was used instead of 1. Obviously, the correlation between *K*p_liver_ and *f*_*u*_ can vary depending on, for example, equations used to predict *K*p_liver_ and the drug charge type.

For the three drugs investigated, similar sets of most influential parameters (i.e., parameters accounting for more than 10% variance of PK outputs) were determined by the three GSA methods exercised (Table S3-5 in the supplement material). However, the parameters ranking and their impact on the specified outputs were different. Generally, more influential parameters were identified by the extended Sobol method than by Morris or Sobol, as a result of parameter correlation.

For quinidine, the importance of parameters correlated with BW and CL_int, CYP3A4_, which are the key influential parameters affecting *C*_max_, *T*_max_, and AUC, was under-estimated by Morris and Sobol, particularly:

1. *V*_liver_, *Q*_pv_, *Q*_HA_, which have moderate to strong correlations with BW in an European Caucasians population used in the simulations.
2. *k*_*a*_, *V*_liver_, intrinsic CL_int_ of CYP2C9 and CYP2E1, which correlates moderately with CL_int_ of CYP3A4 (Fig. 2)

Similar to quinidine, more parameters that are influential were identified by extended Sobol than by Sobol regarding Alprazolam *C*_max_ and AUC. However, for midazolam, the key influential parameters determined by Morris and Sobol methods are nearly the same as extended Sobol for *C*_max_, *T*_max,_ and AUC (Table S5 in the supplement material). This might be due to much shorter half-life (higher clearance) and larger volume of distribution *V*_ss_ of midazolam than alprazolam and quinidine.

Although we expect *f*_*u*_ to affect drug clearance, hence AUC, all three GSA methods suggest that its impact is low for the three drugs. Morris and Sobol methods slightly overestimated the influence of *f*_*u*_ on *T*_max_ and AUC (~ 3 to 6% of variance by Sobol, see Figs. 3, 4, and 5), which was determined as negligible in extended Sobol (< 1% of variance, Figs. 3, 4, and 5). Drug tissue distribution among many other parameters depends on its protein binding. Therefore, it is generally expected to see a strong correlation between *f*_*u*_ and *K*p_liver._ A moderate positive correlation (*r* ~ 0.5) was observed in the simulated population between *f*_*u*_ and *V*_ss_. *K*p_liver_ and *V*_ss_ were predicted using the Rodgers and Rowland method in the Simcyp simulator and showed an insignificant impact on *T*_max_ and AUC. The negligible impact of *V*_ss_, which should be independent of AUC_24h_ or AUC_48h_≈AUC_∞_ (at least in linear cases), was identified correctly by extended Sobol for all three drugs. However, Morris and Sobol overestimated the influence of *V*_ss_ on AUC_24h_ for quinidine and alprazolam.

Although portal vein (*Q*_pv_) blood flow rate is higher than the hepatic artery (*Q*_HA_) blood flow the impact of *Q*_HA_ is calculated to be higher than *Q*_pv_ on *C*_max_ and AUC using the extended Sobol (Table S3-5 in the supplement material). This is mainly due to the fact that the Simcyp simulator simulated population data indicated a slightly stronger correlation between *Q*_HA_ and BW than *Q*_pv_ and BW as shown in Fig. 2. As BW and enzyme intrinsic clearance strongly impact *C*_max_ and AUC, the influence of *Q*_HA_ on these two parameters was also higher. When this correlation was ignored, a higher impact of *Q*_pv_ than *Q*_HA_ on *C*_max_ and AUC was estimated by Sobol and Morris methods (Table S3-5 in the supplement material).

The renal clearance (CL_*R*_) variability simulated using the Simcyp simulator is determined using the simulated subject renal function. In the Simcyp simulator, renal function is assumed to be correlated with the creatinine concentration, which itself is a function of age and gender. However, since CL_*R*_ is small for the three explored drugs, it did not significantly affect the PK parameters.

**Fig. 6:**
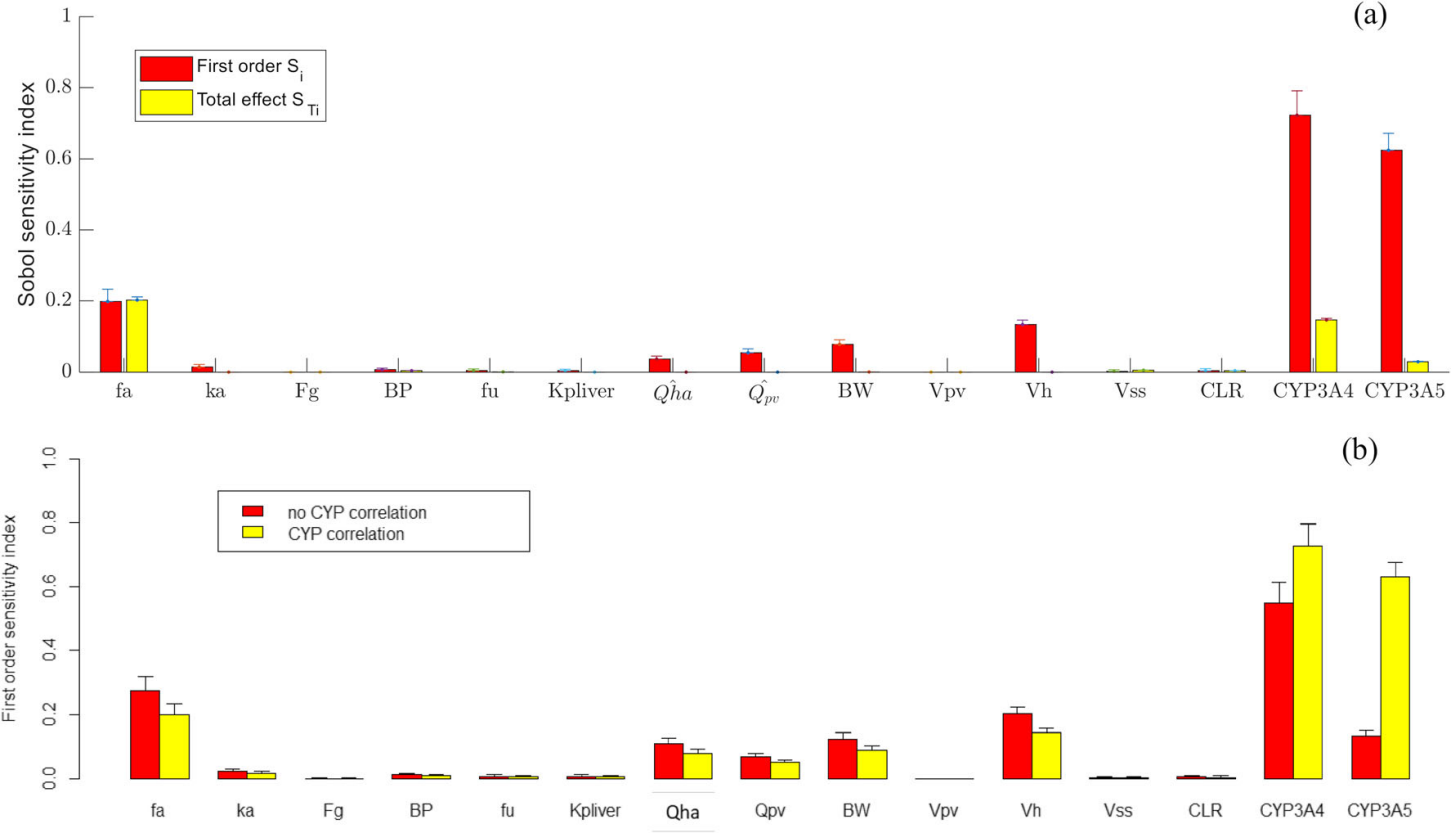
The extended Sobol analysis for AUC_48h_ of alprazolam: **a** first-order and total effect sensitivity indices were reported for model reparametrized using normalised $\hat{Q_{\mathrm{PV}}}$ and $\hat{Q_{\mathrm{HA}}}$, or **b** first-order sensitivity indices were compared for model with or without assumptions that liver CYPs are independent

Furthermore, one should be aware that GSA results are highly dependent on the model explored. For the same underlying physiology or biology, if different mathematical models are used or a model is parameterised in different ways, the influential parameters determined by GSA to explain the same outcome can be different. For example, if one reparametrizes the mPBPK model with normalised blood flow rate; in this case, the hepatic artery and portal vein (to exclude the effect of BW on blood flow) as $\hat{Q_{\mathrm{HA}}}=\frac{Q_{\mathrm{HA}}}{\mathrm{BW}}$ using a lognormal (− 1.24, 1.44e-2) distribution and $\hat{Q_{\mathrm{PV}}}=\frac{Q_{\mathrm{PV}}}{\mathrm{BW}}$ using a lognormal (− 0.11, 1.77e-2) distribution, the impact of $\hat{Q_{\mathrm{PV}}}$ on AUC_48h_ becomes higher than $\hat{Q_{\mathrm{HA}}}$ for alprazolam (Fig. 6a). However, when using the original settings for *Q*_HA_ and *Q*_pv_, the impact of *Q*_HA_ will be higher than *Q*_pv_ due to correlations with BW as explained earlier. Similarly, if one ignores the liver inter-enzyme correlations but keep all other correlations among input parameters the same, the impact of CYPs on PK parameters can change. For example, for alprazolam, the effect of liver CYP3A5 on AUC_48h_ becomes much lower compared to the analysis with consideration of correlation between CYP3A4 and CYP3A5 (Fig. 6b).

Although extended Sobol is a more advanced GSA method than Morris and Sobol, it still has limitations. For instance, it assumes that parameter correlations are linear. The same assumption is made in other GSA methods developed so far for models with correlated parameters (3,9,23–26). In reality, the correlation between parameters can be complicated, and linear correlations may not well represent the relationship between parameters. Therefore, more sophisticated sensitivity algorithms are needed to fully account for realistic correlations. Nevertheless, in comparison with many GSA methods that do not consider parameter correlation, e.g., Morris and Sobol, the extended Sobol method performs well in terms of distinguishing the influential parameters in the mPBPK model for the three investigated drugs as well as test functions. Besides, based on the assessment by Vu-Bac et al. using an example to quantify uncertainty for multiscale modelling of polymer nanocomposites (26), the first-order sensitivity index (*S*_*i*_) of extended sobol is matching the first-order sensitivity index reflecting correlation in other developed methods to handle correlated input parameter, such as the regression-based method by Xu and Gertner (23) and the extension of the matrix combination approach by Most (25). Similarly, the total effect sensitivity index *S*_Ti_ of extended Sobol mainly reflects the influence of uncorrelated components in the input parameters on the metrics of interest. Thus, stronger correlation between certain input parameters gives higher first-order sensitivity index than the total effect index reflected by extended Sobol methods. Therefore, the extended Sobol method can thus provide valuable guidance to correctly recognise influential parameters.

The need to account for correlations in GSA and the availability of data to identify them is somewhat of a circular argument: simple methods unable to consider correlations still give ‘results’ and there seem to be no need for gathering data about them. Our point is precisely that parameter correlations should be investigated carefully first. In the absence of evidence for correlations, simpler GSA methods can be used. Otherwise, more powerful methods are needed to obtain correct results, whether the aim is uncertainty analysis, model development, or study design assessment. Evidence for correlations can come from several sources:

- Mechanistic knowledge of inter-individual variability and parameter dependencies; this was the approach taken here. The Simcyp simulator was used to produce realistically correlated PBPK parameter values, any other PBPK model able to generate such correlated samples could be used: the performance of the different GSA methods in case of correlations were probably not strongly affected by our choice of software. The input correlation values obtained from the Simcyp sample are probably not perfect, but that model is well validated, state of the art and generally trusted (43).
- Statistically modelled relationships between observed parameters (a reasonable modelling choice in the absence of causal explanations).
- Previous model calibration with system-level data. For example, fitting commonly induces correlation between Michaelis-Menten *V*_max_ and *K*_*m*_, by partial identifiability. Unfortunately, the less data we have, the more correlations we are likely to observe, still from identifiability problems. In that case, uncertainty should be modelled with its correlations (using marginals would be unrealistic and would lose information).

Independent measurements of individual parameters, by definition, lead to uncorrelated priors, but this does not mean that those measurements are perfect or even realistic in real-life systems.

Apart from parameter correlation, other essential features affecting the determination of influential parameters in GSA are the distributions and ranges of input parameters. Rather than assuming uniform distributions for model parameters (12,15), the distributions and ranges used in this study were directly obtained from virtual population simulations using the Simcyp simulator, which can realistically simulate human physiology and associated variabilities (43). Since we used physiologically compatible distributions, our results should be more relevant to real-world conditions, rather than being artefacts resulting from arbitrary choices.

Although only three drugs have been investigated using the mPBPK model in the study, the proposed methodology can in principle be applied to any PBPK model to explore the influential parameters of any drug with consideration of parameter correlations.

## Conclusion

We have highlighted some key areas for consideration when applying GSA to identify influential parameters in a model, namely limitations and assumptions of the applied GSA algorithms, assumptions in the investigated physiological or biological model, correlations among model parameters, and distributions or ranges of the parameters of interest. All of these may impact the outcomes, interpretation and application of GSA.

For the three drugs investigated (quinidine, alprazolam, and midazolam), the influential parameters determined by the extended Sobol method, and their ranking, were consistent with the PK properties expected from their physicochemical, plasma/blood binding attributes and the elimination pathways. However, by ignoring correlation among parameters, the Morris and Sobol GSA methods may not correctly identify all important model parameters affecting the model outputs of interest. Particularly, as shown in this study, the effect of *V*_ss_ can be overestimated, and the influence of *V*_liver_ and some enzyme intrinsic clearance/abundance may be underestimated. Almost the same sets and orders of influential parameters have been identified by both the Sobol method and Morris screening, suggesting Morris method can be as informative as the Sobol method to identify the important parameters in the presence of negligible parameter correlations.

Global sensitivity analysis is useful as a general method to assist in model evaluation and feature selection and is particularly valuable to identify influential parameters in models with many input parameters. The GSA algorithms available are developed under various model assumptions, such as linear or non-linear models, monotonic or non-monotonic input-output relationships, and no-correlated or correlated input parameters. It is essential to be fully aware of their limitations to avoid potentially inaccurate conclusions. To the same degree, it is essential, to be fully aware of the model structure and assumptions.

## Electronic supplementary material

ESM 1 (PDF 1431 kb)

## References

[1] Zi Z (2011). Sensitivity analysis approaches applied to systems biology models. IET Syst Biol.

[2] Darwich AS, Neuhoff S, Jamei M, Rostami-Hodjegan A (2010). Interplay of metabolism and transport in determining oral drug absorption and gut wall metabolism: a simulation assessment using the “Advanced Dissolution, Absorption, Metabolism (ADAM)” model. Curr Drug Metab.

[3] Li G, Rabitz H, Yelvington PE, Oluwole OO, Bacon F, Kolb CE, Schoendorf J (2010). Global sensitivity analysis for systems with independent and/or correlated inputs. J Phys Chem A.

[4] Brochot C, Smith TJ, Bois FY (2007). Development of a physiologically based toxicokinetic model for butadiene and four major metabolites in humans: global sensitivity analysis for experimental design issues. Chem Biol Interact.

[5] Davis MJ, Liu W, Sivaramakrishnan R (2017). Global sensitivity analysis with small sample sizes: ordinary least squares approach. J Phys Chem A.

[6] Fenneteau F, Li J, Nekka F (2009). Assessing drug distribution in tissues expressing P-glycoprotein using physiologically based pharmacokinetic modeling: identification of important model parameters through global sensitivity analysis. J Pharmacokinet Pharmacodyn.

[7] Gan Y, Duan Q, Gong W, Tong C, Sun Y, Chu W, Ye A, Miao C, di Z (2014). A comprehensive evaluation of various sensitivity analysis methods: a case study with a hydrological model. Environ Model Softw.

[8] Gueorguieva I, Nestorov IA, Rowland M (2006). Reducing whole body physiologically based pharmacokinetic models using global sensitivity analysis: diazepam case study. J Pharmacokinet Pharmacodyn.

[9] Kucherenko S, Tarantola S, Annoni P (2012). Estimation of global sensitivity indices for models with dependent variables. Comput Phys Commun.

[10] Lumen A, McNally K, George N, Fisher JW, Loizou GD (2015). Quantitative global sensitivity analysis of a biologically based dose-response pregnancy model for the thyroid endocrine system. Front Pharmacol.

[11] Marino S, Hogue IB, Ray CJ, Kirschner DE (2008). A methodology for performing global uncertainty and sensitivity analysis in systems biology. J Theor Biol.

[12] McNally K, Cotton R, Loizou GD (2011). A workflow for global sensitivity analysis of PBPK models. Front Pharmacol.

[13] Sumner T, Shephard E, Bogle ID (2012). A methodology for global-sensitivity analysis of time-dependent outputs in systems biology modelling. J R Soc Interface.

[14] Reuter U, Liebscher M. Global sensitivity analysis in view of nonlinear structural behavior. Proceedings of the 7th LS-DYNA Forum, Bamberg. 2008; F-I-02.

[15] Scherholz ML, Forder J, Androulakis IP (2018). A framework for 2-stage global sensitivity analysis of GastroPlus compartmental models. J Pharmacokinet Pharmacodyn.

[16] McNally K, Cotton R, Cocker J, Jones K, Bartels M, Rick D (2012). Reconstruction of exposure to m-xylene from human biomonitoring data using PBPK modelling, Bayesian inference, and Markov chain Monte Carlo simulation. J Toxicol.

[17] Iooss B, Lemaître P, Meloni C, Dellino G (2015). A review on global sensitivity analysis methods. Uncertainty management in simulation-optimization of complex systems: algorithms and applications.

[18] Saltelli A, Ratto M, Andres T, Campolongo F, Cariboni J, Gatelli D, et al. Global sensitivity analysis. The Primer. Wiley; 2008.

[19] Zhang XY, Trame MN, Lesko LJ, Schmidt S (2015). Sobol sensitivity analysis: a tool to guide the development and evaluation of systems pharmacology models. CPT Pharmacometrics Syst Pharmacol.

[20] Wagener T, Kollat J (2007). Numerical and visual evaluation of hydrological and environmental models using the Monte Carlo analysis toolbox. Environ Model Softw.

[21] Pianosi F, Wagener T (2015). A simple and efficient method for global sensitivity analysis based on cumulative distribution functions. Environ Model Softw.

[22] Wagener T, McIntyre N, Lees MJ, Wheater HS, Gupta HV (2003). Towards reduced uncertainty in conceptual rainfall-runoff modelling: dynamic identifiability analysis. Hydrol Process.

[23] Xu C, Gertner G (2007). Extending a global sensitivity analysis technique to models with correlated parameters. Comput Stat Data Anal.

[24] Xu C, Gertner GZ (2008). Uncertainty and sensitivity analysis for models with correlated parameters. Reliab Eng Syst Saf.

[25] Most T, editor. Variance-based sensitivity analysis in the presence of correlated input variables. Proceedings 5th International Conference on Reliable Engineering Computing (REC); 2012; Brno, Czech Republic.

[26] Vu-Bac N, Rafiee R, Zhuang X, Lahmer T, Rabczuk T (2015). Uncertainty quantification for multiscale modeling of polymer nanocomposites with correlated parameters. Compos B Eng.

[27] Sobol′ IM. Global sensitivity indices for nonlinear mathematical models and their Monte Carlo estimates. Math Comput Simul. 2001;55:271–80.

[28] Wentworth MT, Smith RC, Banks HT (2016). Parameter selection and verification techniques based on global sensitivity analysis illustrated for an HIV model. SIAM-ASA J Uncertain.

[29] Bilal N, editor. Implementation of Sobol’s method of global sensitivity analysis to a compressor simulation model. 22nd International Compressor Engineering Conference; 2014; West Lafayette, Indiana.

[30] Sobol IM (1993). Sensitivity estimates for nonlinear mathematical models. Math Modelling Comput Exp.

[31] Homma T, Saltelli A (1996). Importance measures in global sensitivity analysis of nonlinear models. Reliab Eng Syst Saf.

[32] Saltelli A, Annoni P, Azzini I, Campolongo F, Ratto M, Tarantola S (2010). Variance based sensitivity analysis of model output. Design and estimator for the total sensitivity index. Comput Phys Commun.

[33] Beaudouin R, Goussen B, Piccini B, Augustine S, Devillers J, Brion F, Péry ARR (2015). An individual-based model of zebrafish population dynamics accounting for energy dynamics. PLoS One.

[34] Saltelli A, Tarantola S, Campolongo F, Ratto M (2004). Sensitivity analysis in practice: a guide to assessing scientific models.

[35] Rowland Yeo K, Jamei M, Yang J, Tucker GT, Rostami-Hodjegan A (2010). Physiologically based mechanistic modelling to predict complex drug–drug interactions involving simultaneous competitive and time-dependent enzyme inhibition by parent compound and its metabolite in both liver and gut—the effect of diltiazem on the time-course of exposure to triazolam. Eur J Pharm Sci.

[36] Campbell TJ (1992). Subclassification of class I antiarrhythmic drugs: enhanced relevance after CAST. Cardiovasc Drugs Ther.

[37] Ait-Daoud N, Hamby AS, Sharma S, Blevins D (2018). A review of alprazolam use, misuse, and withdrawal. J Addict Med.

[38] Brown EN, Purdon PL, Van Dort CJ (2011). General anesthesia and altered states of arousal: a systems neuroscience analysis. Annu Rev Neurosci.

[39] Pang W, Lin RMH, Lin ML, Chen YO, Lin JC, Yang CH (2018). Midazolam-induced unexpected monoparesis: not contraindicated for ambulatory general anesthesia. Ambul Surg.

[40] Cote CJ, Cohen IT, Suresh S, Rabb M, Rose JB, Weldon BC (2002). A comparison of three doses of a commercially prepared oral midazolam syrup in children. Anesth Analg.

[41] Fenneteau F, Turgeon J, Couture L, Michaud V, Li J, Nekka F (2009). Assessing drug distribution in tissues expressing P-glycoprotein through physiologically based pharmacokinetic modeling: model structure and parameters determination. Theor Biol Med Model.

[42] Melillo N, Aarons L, Magni P, Darwich AS (2019). Variance based global sensitivity analysis of physiologically based pharmacokinetic absorption models for BCS I-IV drugs. J Pharmacokinet Pharmacodyn.

[43] Jamei M, Dickinson GL, Rostami-Hodjegan A (2009). A framework for assessing inter-individual variability in pharmacokinetics using virtual human populations and integrating general knowledge of physical chemistry, biology, anatomy, physiology and genetics: a tale of ‘bottom-up’ vs ‘top-down’ recognition of covariates. Drug Metab Pharmacokinet.

