# Supplementary material for: Considerations and Caveats when Applying Global Sensitivity Analysis Methods to Physiologically Based Pharmacokinetic Models
Source: AAPS J. 2020 Jul 17;22(5):93. doi: 10.1208/s12248-020-00480-x (PMC7367914; doi:10.1208/s12248-020-00480-x)
Supplement: Supplementary file 1 — (PDF 1431 kb) [file 12248_2020_480_MOESM1_ESM.pdf]

Appendix A1

Review of GSA methods

GSA methods for models with non-correlated variables

1) Elementary effect method

The most widely used elementary effect method is Morris screening. Although Morris method is efficient to be implemented as it requires relatively low computational cost, it can only provide qualitatively measure of the sensitivity as an overall measure of the interactions was calculated. Moreover, negative elements of the elementary effects may be observed for a non-monotonic model. Therefore, the mean measured elementary effects are not always reliable when ranking the importance of the variables [1]. The accuracy of Morris method will rely on the choice of the levels and the number of trajectory when sampling.

2) Variance-based method

The variance-based methods is to quantify the amount of variance that each input variable contributes with on the unconditional variance of the output. Sobol, Fourier amplitude sensitivity test (FAST), extended Fourier amplitude sensitivity test (eFAST) are most commonly adopted variance-based GSA methods in engineering, biology, and clinical studies. The Sobol method does not assume the relationship between the model inputs and outputs [1]. It decomposes the variance of the model output function into summands of variances in combinations of input variables in increasing dimensionality [2]. Theoretically, Sobol method can compute sensitivity indices of any orders. In practice, the computational cost can be expensive for indices higher than second-order [3]. Similar to Sobol method, FAST is a modified variance-based sensitivity analysis method based on Fourier transformation and analysis. Since Fourier analysis measures the strength of each parameter's frequency in the model output, the model's sensitivity to any input parameter can be estimated through how strongly the parameter's frequency propagates from input through the model to the output [4]. Classic FAST method can only estimation first order sensitivity index for each parameter. Saltelli et al. [5] extended FAST to eFAST by introducing a new transformation matrix, which allows to quantify total effect.

The variance-based sensitivity analysis approaches (e.g. Sobol, FAST, HDMR methods) were generally recommended [6], since they are independent on model linearity or monotonicity, 2) are capable to obtain the impact of the full range of each input parameter, and 3) allow to evaluate accurately the interaction effects among input variables. However it is well-known that the variance-based approaches requires high computational cost, particularly for models with large amount of variables [1]. Although the variance-based method can estimate main, interaction and total effects precisely with enough computational resource, its accuracy still depends on the true value of indices, e.g. variables with greater effect will have better evaluations.

3) Regression and correlation based method

Regression and Correlation based GSA models, such as standardised regression correlation (SRC), partial correlation coefficient (PCC), and partial ranked correlation coefficient (PRCC), are also popular approaches [6, 7]. SRC and PCC were not suitable for non-linear situation or non-monic problems as they were developed based on linear and monotonic assumption [1, 3]. PRCC is robust for nonlinear problem, but still depends on a monotonic assumption between inputs and outputs. Hence it will be inaccuracy when non-monotonicities in the model are present [4].

4) Method based on Monte Carlo filtering

Multi-parametric sensitivity analysis (MPSA) is one type of GSA method based on Monte-Carlo filtering, which evaluates the parameter sensitivity based on Kolmogorov–Smirnov statistics by classifying the parameter sets [1]. The overall impact of the input variables on the model output can be investigated by MPSA method. However, the estimation can be subjective as an acceptable threshold and a proper distribution of variables are required, which can be subject to high inter-observer variability.

5) Meta model-based methods

Meta-modelling method is to analyse the impact of input parameter on model output by replacing the original models with statistical or experimental design methods [7, 8].

GSA method for models with correlated input variables

Although variance-based method is robust to nonlinear and non-monotonic system, it does assume the input space are not correlated. Recently a few studies have been proposed to extend the variance-based method to cope with correlated input variables [9-13].

1) Extension of regression method

Xu and Gertner developed a variable decomposition method based on regression by considering the correlation among input variables, which works well when there is an approximately linear relationship between response and inputs [13]. Three indices corresponding to uncorrelated, correlated, and total (=uncorrelated + correlated) effect will be derived to assess parameter importance.

2) Extension of variance-based method

Extension of FAST

An extension of FAST was developed by Xu and Gertner [12] to models with correlated variables. The characteristic frequency of a parameter is exploited to capture both the uncertainties of the parameter itself and the dependent variations of other variables. Similar to FAST, only main effect index can be quantified.

Extension of HDMR

Li et al [10] established a new framework of global sensitivity analysis, named as structural and correlative sensitivity analysis (SCSA), for models with independent and/or correlated inputs. SCSA relies on covariance decomposition of the unconditional variance of the output, and split the contribution of an input to structural and correlative parts. Three indices will be derived corresponding to structural, correlative, and total (= structural + correlative) effect respectively. When inputs are independent this proposed framework will reduce to a random sampling-high dimensional model representation (RS-HDMR) method.

Also, Zuniga et al. [14] extended the random sampling (RS) or Quasi Monte-Carlo sampling (QMC)-HDMR methods to coupe with model with dependent variables. This method was claimed to be more efficient than the extended Sobol method by Kucherenko et al. [9] in evaluations of main effect.

Extension of Sobol method (exSobol)

An extended Sobol method was proposed by Most et al. [11] assuming a linear correlation between model input variables. This method divided the contribution of an input to variance into uncorrelated (independent) and correlative parts, hence 4 sensitivity indices would be available, e.g. uncorrelated and correlated first-order and total indices. Similarly, Mara et al. [15] developed a new variance-based sensitivity analysis method based on the Gram-Schmidt decorrelation procedure and the polynomial chaos expansion (PCE). A set of new sensitivity indices, called full sensitivity indices, can be derived using the new set of independent variables after decorrelating the input variables [16]. Besides, Kucherenko et al. [9] developed a copula-based method to calculate the main (first-order) and total effect analogous to standard Sobol indices. This method would need a priori knowledge of parameter probability distribution, but does not use surrogate models, data-fitting procedures or orthogonalization of the input factor space.

Appendix A2

Evaluation of GSA methods on test functions

1. GSA analysis for model with non-correlated inputs

1.1 Test function 1 - Ishigami-Homma function

Ishigami-Homma function is a non-linear and non-monic function commonly used in the Global Sensitivity Analysis [6].

$$Y = \sin(x_1) + a\times\sin^2(x_2) + b\times x_3^4\times\sin(x_1) \quad (1)$$

where x_i ~ [-π, π] for all i = 1, 2, 3. a and b are constants, e.g. a = 7 and b = 0.05 [17].

The analytical solutions for first and total order indices are

First order:

$S_i(x_1) = (1/2+ b\times\pi^4/5+b^2\times\pi^8/50)/V$;

$S_i(x_2) = (a^2/8)/V$;

$S_i(x_3) = 0$;

Total effect:

$S_{Ti}(x_1) = S_i(x_1) + (b^2\times\pi^8/18- b^2\times\pi^8/50)/V$;

$S_{Ti}(x_2) = S_i(x_2)$;

$S_{Ti}(x_3) = (b^2\times\pi^8/18- b^2\times\pi^8/50)/V$;

where variance $V=1/2+a^2/8+b\times\pi^4/5+b^2\times\pi^8/18$;

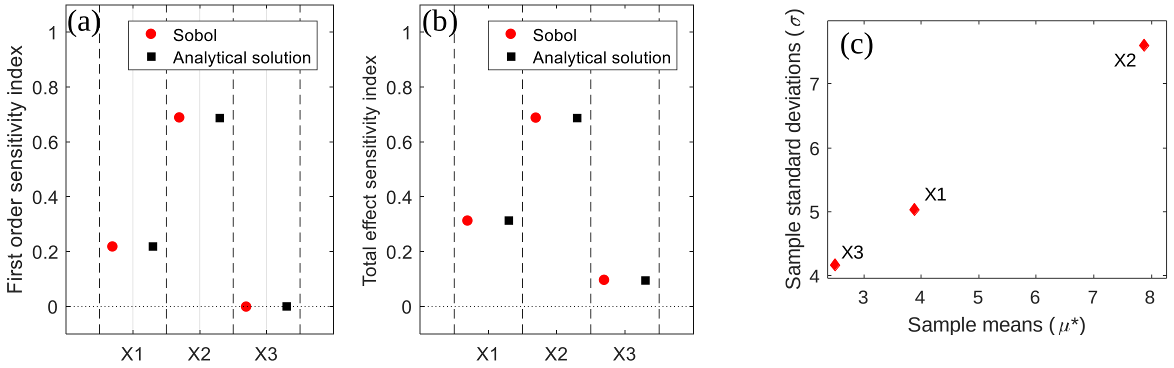

Figure S1. Comparison of estimated sensitivity indices to analytical solutions for Ishigami-Homma function: random sampling N=2000 for Sobol indies (a and b); Morris screening with 4 levels and 10 sampling (c).

Fig. S1 shows that the estimated Sobol sensitivity indices agree well with the analytical solutions for Ishigami-Homma function. Also the parameters ranked by Morris method matches the parameter importance ordered according to their analytical values (Fig. S1c). Small negative first-order index of Sobol indices can occur, due to the random sampling for Monte-Carlo estimation, which was also observed in other studies [18].

1.2 Test function 2 – Sobol g-function

Sobol g-function is another function widely used in the Global Sensitivity Analysis, particularly due to its strong nonlinearity and non-monotonicity [6]:

$$Y = \prod_{i=1}^{k} g_i\,(X_i) \text{ and } g_i\,(X_i) = \frac{|4X_i - 2| + a_i}{1 + a_i} \qquad (2)$$

where a_i are non-negative parameters, and X_i ~ [0, 1], for all i = 1, …, k.

The analytical solutions for first and total order indices are

First order:

$$V_i = \frac{1}{3(1+a_i)^2}$$

$$V = \prod_{i=1}^{k}(1+V_i) - 1$$

$$S_i\,(X_i) = V_i / V$$

Total order:

$$V_{Ti} = V_i \prod_{j \neq i}(1+V_j)$$

$$S_{Ti}(X_i) = V_{Ti} / V$$

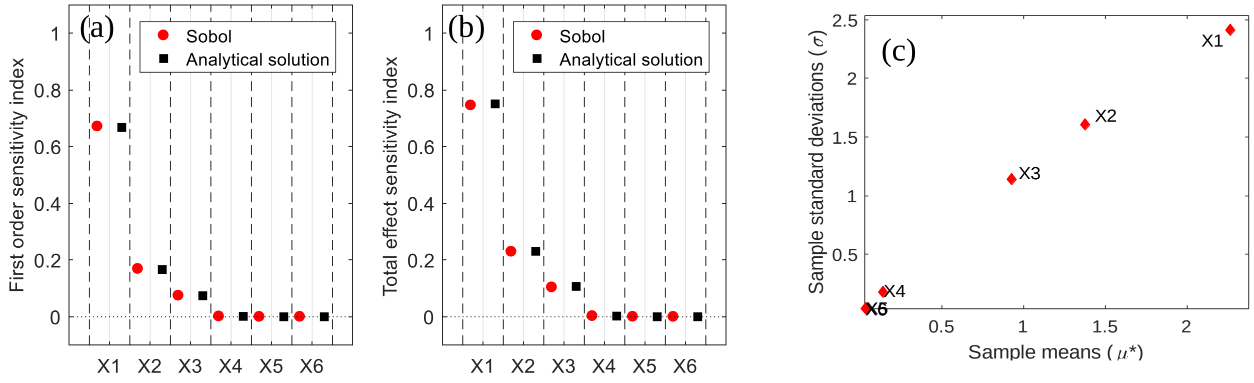

Figure S2. Comparison of estimated sensitivity indices to analytical solutions for g-function: random sampling N=2000 for Sobol indices (a and b); Morris screening with 4 levels and 10 sampling (c). The constants a_i were set as a_1=0, a_2=1, a_3=2, a_4=19, and a_5=a_6=99 [19].

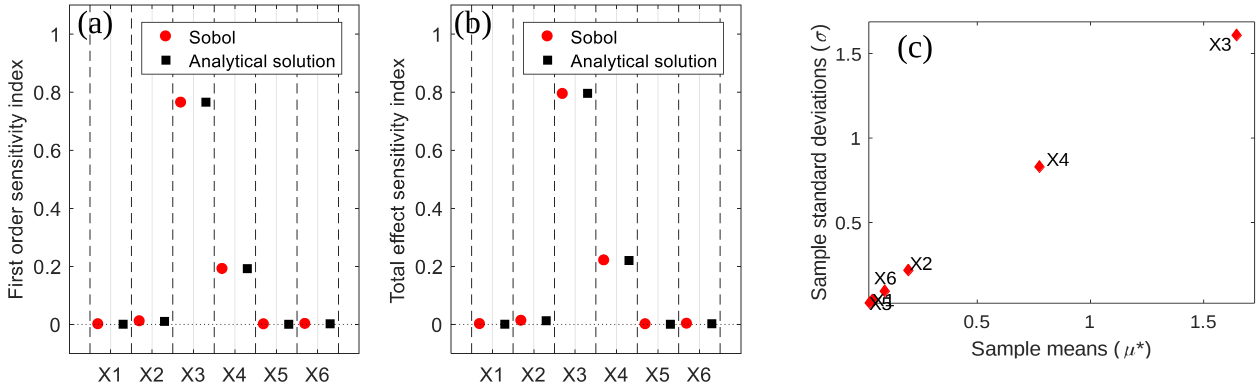

Figure S3. Comparison of estimated sensitivity indices to analytical solutions for g-function: random sampling N=2000 for Sobol indices (a and b). Morris method with 4 levels and 10 sampling (c). Constants a_i (i = 1 to 6) = (78, 12, 0.5, 2, 97, 33)[6].

Similar to the analysis for Ishigami-Homma function, the estimated Sobol sensitivity indices are in good agreement with their analytical solutions for non-linear g-function (Fig. S2 and S3). The parameters ordered by Morris method also agrees well with the parameter importance graded based on their analytical values (Fig. S2 and S3).

2. GSA analysis for model with correlated parameter

2.1 Test function 3 - $Y = x_1 + x_2 + x_3$ (3)

Simple linear function was adopted from Kucherenko et al. [9] to evaluate the impact of correlation on estimation of sensitivity indices. Input variables x_i have normal distribution with mean μ=0 and covariance matrix as

$$\Sigma = \begin{pmatrix} 1 & 0 & 0 \\ 0 & 1 & \rho\sigma \\ 0 & \rho\sigma & \sigma^2 \end{pmatrix}$$

where ρ is the correlation coefficient, and σ is the standard deviation. The analytical solution for first and total order sensitivity indices were given as

$$S_1 = \frac{1}{2+\sigma^2+2\rho\sigma}, \quad S_1^T = \frac{1}{2+\sigma^2+2\rho\sigma}$$

$$S_2 = \frac{(1+\rho\sigma)^2}{2+\sigma^2+2\rho\sigma}, \quad S_2^T = \frac{1-\rho^2}{2+\sigma^2+2\rho\sigma}$$

$$S_3 = \frac{(\sigma+\rho)^2}{2+\sigma^2+2\rho\sigma}, \quad S_3^T = \frac{\sigma^2(1-\rho^2)}{2+\sigma^2+2\rho\sigma}$$

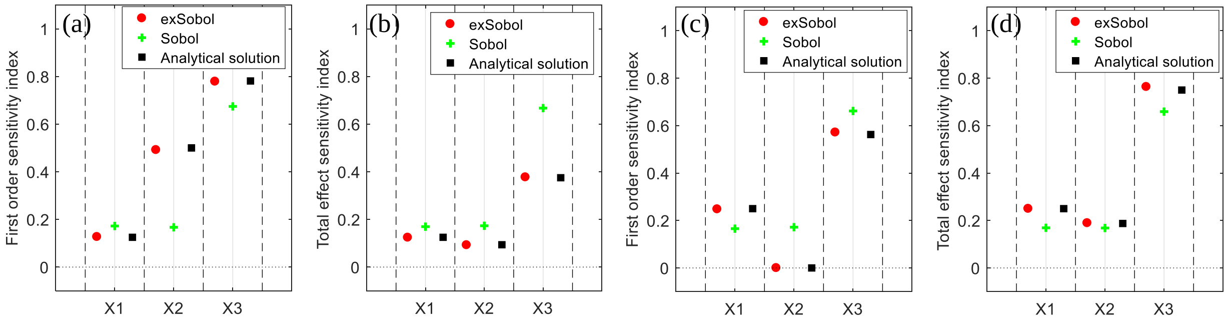

Figure S4. Comparison of estimated sensitivity indices to analytical solutions for test function 3: Correlation coefficient ρ_{23}=0.5, σ=2 (a and b); Correlation coefficient ρ_{23}=-0.5, σ=2 (c and d).

Overall, extended Sobol recovered both main and total effects well for test function 3 (Fig. S4). However, the accuracy of Sobol, developed for models with non-correlated inputs, will be compromised by the presence of correlation among input parameters.

2.2 *Test function 4 - Ishigami-Homma function*

The non-linear and non-monotonic Ishigami-Homma function in section 1.1 was adopted for validation of extended Sobol method with uniform distributed inputs $x_i \in [-\pi, \pi]$, and a=7 and b=0.1 [9]. The estimated first order and total order sensitivity indices agree well with results presented by Kucherenko et al. [9] with random sample size N = 2^{13} (Fig. S5). Minor differences would be expected due to the random sampling of input parameters.

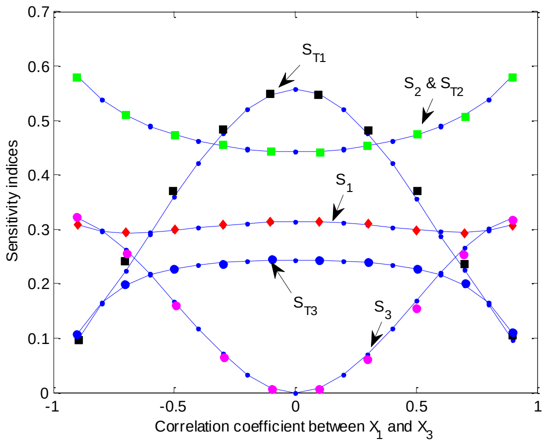

Figure S5. Change of the first order and total order sensitivity indices of the Ishigami function at different correlation values ρ_{13}. Closed markers are estimations by Kucherenko et al. [9], dot line represents results of this work.

Appendix A3

Results of Morris method for Quinidine, Alprazolam, and Midazolam

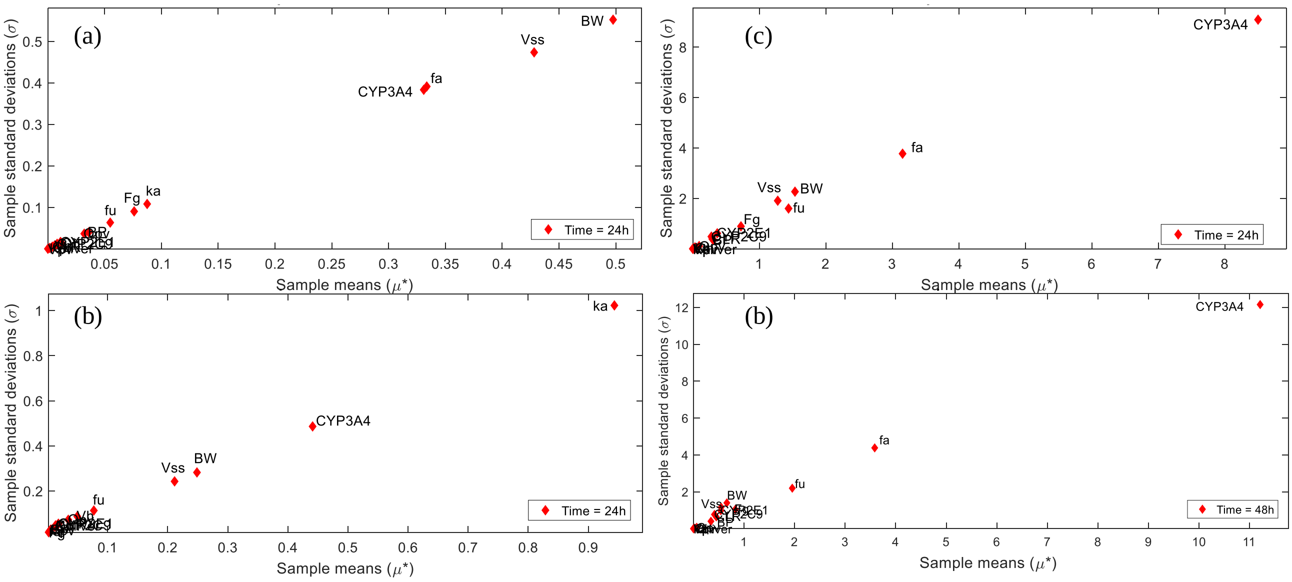

Figure S6, Morris measures for Quinidine (a) C_{max}, (b) T_{max}, (c) AUC_{24h}, and (d) AUC_{48h}

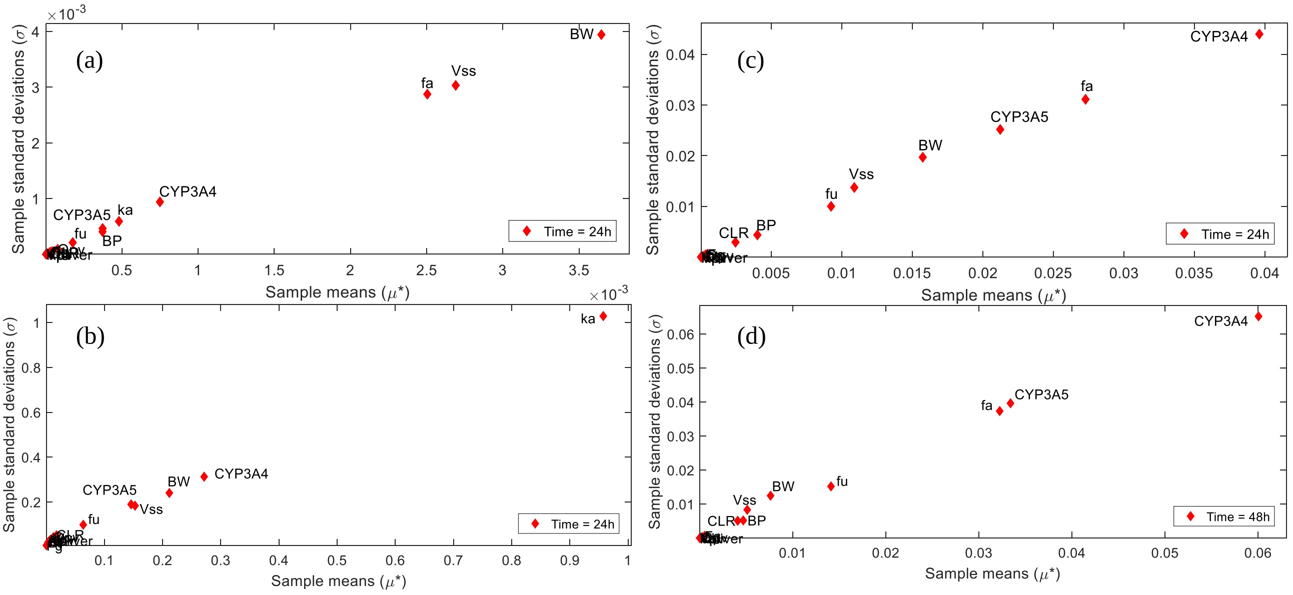

Figure S7, Morris measures for Alprazolam (a) C_{max}, (b) T_{max}, (c) AUC_{24h}, and (d) AUC_{48h}

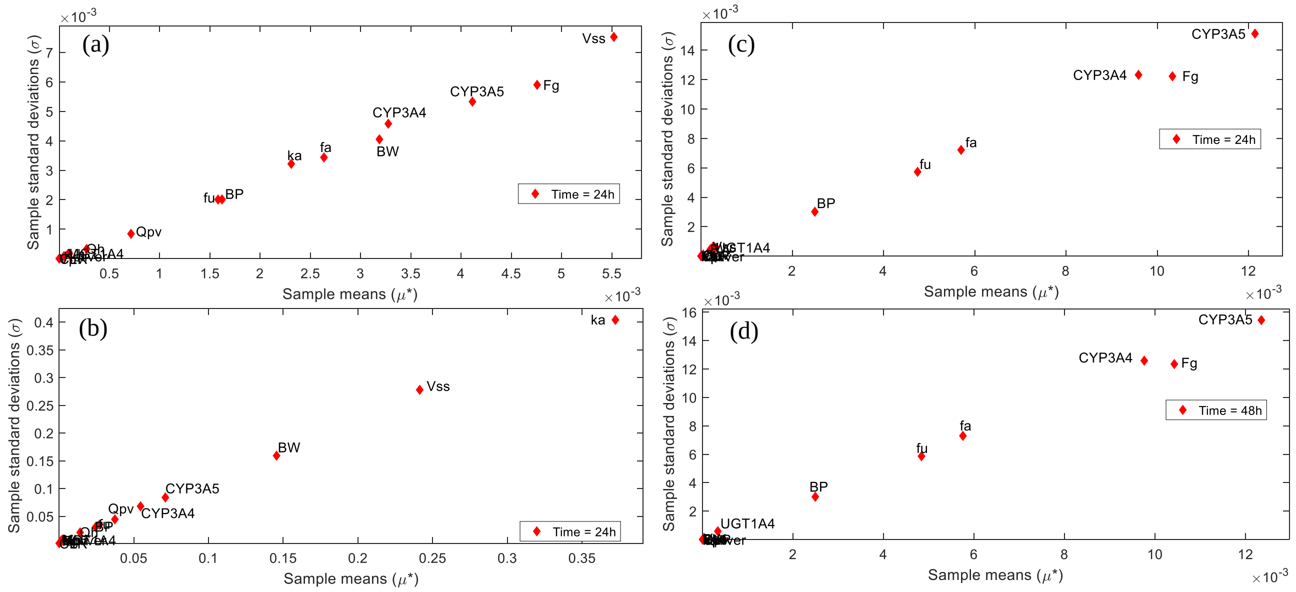

Figure S8, Morris measures for Midazolam (a) C_{max}, (b) T_{max}, (c) AUC_{24h}, and (d) AUC_{48h}

Appendix 4

Morris screening method

Briefly, each of the n parameters of interest, X_i (i in 1, …, n), is first rescaled to fall between 0 and 1, and discretized to form a grid of l equally spaced values (levels) of X_i. The grid therefore contains values $\frac{1}{l-1}, \frac{2}{l-1}, \ldots, \frac{l-2}{l-1}$, 1. The elementary effect of a given parameter, X_i, is calculated by a simple difference formula as shown below [1, 6, 20]:

$$d_i(X) = \frac{f(X_1, X_2, X_3, \cdots, X_i + \Delta, X_{i+1}, \cdots, X_n) - f(X)}{\Delta} \quad (1)$$

where Δ is the difference (step size) between two random grid values of X_i chosen to evaluate f. A series of r sample points are randomly drawn in the parameter space of interest. Using even values for l and choosing $\Delta = l/[2(l-1)]$ in a suitable sampling scheme guarantees equal sampling probability for each grid parameter value [20]. For the sth sample, the elementary effect of the ith parameter is determined as follows:

$$d_i^s = \frac{f(X^s + \Delta) - f(X^s)}{\Delta} \quad (2)$$

For a total of r sample points, the Morris indices for the parameter X_i are defined as:

$$\mu_i = \frac{1}{r}\Sigma_{s=1}^{r} d_i^s \quad (3)$$

$$\mu_i^* = \frac{1}{r}\Sigma_{s=1}^{r} |d_i^s| \quad (4)$$

$$\sigma_i^2 = \frac{1}{r-1}\Sigma_{s=1}^{r} (d_i^s - \mu_i)^2 \quad (5)$$

A high μ or μ* indicates a parameter with an important overall influence on the model outputs; a high σ indicates either a parameter interacting with other factors or that its effect is non-linear. The magnitudes of μ and σ for each model parameter are relative to the others and have no absolute meaning [21].

Sobol method

Sobol method is a variance-based type GSA method, which decompose the variance of the model outputs into sums of variances for combinations of input parameters of increasing dimensionality [2]. Although there is no assumption about the relationship between the model inputs and outputs in variance-based GSA methods, they do assume that the input parameters are independent. Generally, when using Sobol, three sensitivity indices are calculated to determine the importance of input parameters:

- A first-order "main effect" sensitivity index evaluating only the main influence of each parameter without considering the interaction with others;
- A "total-effect" sensitivity index to assess the impact of each parameter including all possible interactions with others;

- An "interaction" index, which is the difference between total effect and main effect, representing only the contribution of parameters interactions.

Details of the derivation of Sobol sensitivity indices are explained in [18, 22-25].

Extended Sobol method

The GSA method proposed by Kucherenko et al. [9] can consider models where the input parameters are correlated. The main (first-order) and total effect sensitivity indices, analogous to standard Sobol indices, are calculated using a copula-based method. Briefly, for a function $f(x_1,..., x_n)$ of a random vector $(x_1, \dots, x_n)$ defined on R^n (real values of dimension n) with a finite variance, the variable set $x=\{x_1,..., x_n\}$ can be partitioned as two subsets as $y = \{x_1,..., xi_s\}$, $1 \leq i_s < n$, and a complementary subset $z = \{x_{is+1},..., x_n\}$, so that x={y,z}. The total variance V can be decomposed as [9]:

$$V = V_y\left[E_z\left(f(y, \bar{z})\right)\right] + E_y\left[V_z\left(f(y, \bar{z})\right)\right] \quad (6)$$

where the random vector $\bar{z}$ is a sample from the conditional distribution $p(z|y)$ and E is the expectation function over f with respect with its subscript. The corresponding first-order effect index of the subset y is defined as:

$$S_y = \frac{V_y\left[E_z\left(f(y,\bar{z})\right)\right]}{V} \quad (7)$$

While the total-effect index of the subset y is:

$$S_y^T = \frac{E_y\left[V_z\left(f(y,\bar{z})\right)\right]}{V} \quad (8)$$

Collectively, S_y and S_y^T in the case of *independent variables* are called Sobol's Indices.

The first order effect S_y can be estimated as [9]:

$$S_y = \frac{1}{V}\left\{\int_{R^s} p(y)\left[\int_{R^{n-s}} f(y,\bar{z})p(y,\bar{z}|y)d\bar{z} \times \int_{R^{n-s}} f\left(y,\bar{z}'\right)p\left(y,\bar{z}'\middle|y\right)d\bar{z}'\right]dy - f_0^2\right\} \quad (9)$$

$$f_0^2 = \left[\int_{R^n} f(y,z)p(y,z)dydz\right]^2 \quad (10)$$

Here p(y) is the marginal distribution function of y, the random vector $(y,\bar{z}')$ is a second random vector drawn from the conditional probability distribution $p(y, z|y)$, and (y, z) is a sample from the joint distribution p(y, z).

The total order index is given as:

$$S_y^T = \frac{1}{2V}\int_{R^{n+s}}[f(y,z) - f(y',z)]^2\, p(y,z)p(y',z|z)dydy'dz \quad (11)$$

Correlation analysis can be adopted to provide *a priori* knowledge of probability distribution function $p(y, z)$ required by the extended Sobol method [16]. If a linear correlation between two input parameters is assumed, say, X_i and X_j, the correlation coefficient, ρ_{ij}, is calculated as follows:

$$\rho_{ij} = \frac{Cov(X_i, X_j)}{\sigma_{x_i}\sigma_{x_j}} \quad (12)$$

The covariance $Cov(X_i, X_j)$ between X_i, X_j is:

$$Cov(X_i, X_j) = E\left[\left(X_i - \overline{X_i}\right)\left(X_j - \overline{X_j}\right)\right] \quad (13)$$

where $\bar{X}_i$, $\bar{X}_j$ and σ_{Xi}, σ_{Xj} are means and standard deviations of X_i and X_j, respectively.

If the correlation matrix is the identity matrix (hence if there are no correlations), the extended Sobol will be reduced to a simple Sobol method using the Jensen–Sobol' formula [9]. *I.e.*, the conditional distributions in equation 14 is reduced to a marginal distribution: $p(y, z|y) = p(z)$. The total effect in equation 14 is reduced to:

$$S_y^T = \frac{1}{2V}\int_{R^{n+s}}[f(y,z) - f(y',z)]^2\, p(y)p(y')p(z)dydy'dz \quad (14)$$

Appendix 5

Table S1. Key features of GSA methods for models with non-correlated input parameters

Elementary effect method, *e.g. Morris screening.*	• *Semi-quantitative analysis*<br>• *Fast, but results may not be robust*<br>• *Can handle non-linear and non-monotonic problems*
Variance-based method, *e.g. Sobol, FAST, eFAST, other HDMR methods.*	• *Decomposition of variance*<br>• *Robust for non-linear or non-monotonic problems*<br>• *Require* a priori *knowledge of parameter probability distribution*<br>• *Relative high computation cost*
Regression and correlation based method, *e.g. SRC, PCC, and PRCC.*	• *Linear and monotonic model assumption (SRC, PCC)*<br>• *Non-linear but monotonic model (PRCC)*
Method based on Monte Carlo filtering, *e.g. Multi-parametric sensitivity analysis (MPSA)*	• *Can be subjective since a threshold and distribution of parameters are required to guarantee the Monte-Carlo simulations generate sufficient 'acceptable' hits for analysis, which can be subject to high inter-observer variability*
Meta model-based methods	• *Replacing the original models with statistical or experimental design methods*<br>• *Accuracy subject to the robust of surrogate model*

Table S2. Key features of GSA methods for model with correlated input parameters

Extension of regression method			*Suitable for linear system*
Extension of Variance-Based Methods	Xu's extension of FAST		*Similar to FAST, only main effect index can be quantified*
	Extension of HDMR, *e.g. structural and correlative sensitivity analysis (SCSA)*		• *Covariance decomposition of the unconditional variance of the output*<br>• *Contributions of an input are split to structural and correlative parts.*<br>• *Three indices corresponding to structural, correlative, and total (= structural + correlative) effect respectively.*
	Extension of Sobl	*extended Sobol*	• *Copula-based method to calculate the main (first-order) and total effect analogous to standard Sobol indices.*<br>• *Requires* a priori *knowledge of parameter probability distribution*<br>• *Does not use surrogate models, data-fitting procedures or orthogonalization of the input factor space*
		Mara's method	• *Based on the Gram-Schmidt decorrelation procedure and the polynomial chaos expansion (PCE).*<br>• *A set of new sensitivity indices derived after de-correlating the input parameters.*
		Most's method	• *Linear correlation is assumed among model input parameters.*<br>• *The contribution of an input to variance will be split into uncorrelated (independent) and correlative parts.*

Table S3. Ranked influential parameters for Quinidine

	C_{max}			T_{max}			AUC_{24h}			AUC_{48h}		
	Morris	Sobol	extended Sobol	Morris	Sobol	extended Sobol	Morris	Sobol	extended Sobol	Morris	Sobol	extended Sobol
f_a	3	3	1	15			2	2	2	2	2	2
k_a	5	5		1	1	1	13		9	13		9
F_g	6		10	16			6		10	5		10
BP	8		11	13		8	10		_	10		
f_u	7			5	5		5	4	_	3	3	
K_{pliver}	15			11			15		_	16		
Q_{HA}	11		3	12			12		6	12		6
Q_{PV}	9		8	7			11		8	11		8
BW	1	1	4	3	3	7	3	3	7	4		7
V_{pv}	16			14			16			15		
V_{liver}	13		5	6		3	14		3	14		3
V_{ss}	2	2	9	4	4	6	4	5		6		
CL_R	14			9			9			9		
$CL_{int,CYP2E1}$	10		7	8		5	7		5	7		5
$CL_{int,CYP2C9}$	12		6	10		4	8		4	8		4
$CL_{int,CYP3A4}$	4	4	2	2	2	2	1	1	1	1	1	1

*For the extended Sobol and Sobol, numbers underlined indicate input parameters with sensitivity index >0.01 and <0.1, i.e. parameters having only moderate impact on the outputs.

+For Morris screening, all input parameters were ranked based on its GI metric.

Table S4. Ranked influential parameters for Alprazolam

	C_{max}			T_{max}			AUC_{24h}			AUC_{48h}		
	Morris	Sobol	extended Sobol	Morris	Sobol	extended Sobol	Morris	Sobol	extended Sobol	Morris	Sobol	extended Sobol
f_a	3	3	2	13			2	2	3	3	3	3
k_a	5			1	1	1	11		9	12		8
F_g	10			15			9			9		
BP	7			14			7			7		
f_u	8		10	6	6		6	6		4	4	
K_{pliver}	14		9	11			15			15		
Q_{HA}	11		4	12			12		6	11		6
Q_{PV}	9		5	8			10		7	10		7
BW	1	1	3	3	3	6	4	4	5	5	5	5
V_{pv}	15			10			13			13		
V_{liver}	13		6	9		4	14		4	14		4
V_{ss}	2	2	1	5	4	5	5	5	8	6		
CL_R	12			7			8			8		
$CL_{int,CYP3A4}$	4	4	8	2	2	2	1	1	1	1	1	1
$CL_{int,CYP3A5}$	6		7	4	5	3	3	3	2	2	2	2

*For the extended Sobol and Sobol, numbers underlined indicate input parameters with sensitivity index >0.01 and <0.1, i.e. parameters having a only moderate impact on the outputs.

+For Morris screening, all input parameters were ranked based on its GI metric.

Table S5. Ranked influential parameters for Midazolam

	C_{max}			T_{max}			AUC_{24h}			AUC_{48h}		
	Morris	Sobol	extended Sobol	Morris	Sobol	extended Sobol	Morris	Sobol	extended Sobol	Morris	Sobol	extended Sobol
f_a	6	6	9	14			4	4	6	4	4	6
k_a	7	7	14	1	1	1	12		11	12		11
F_g	2	2	2	15			2	2	3	2	2	3
BP	8	8	11	8		7	6	6	10	6	6	10
f_u	9	9	12	7			5	5		5	5	
K_{pliver}	14		13	12			15			15		
Q_{HA}	11		8	9			11		8	13		8
Q_{PV}	10		10	6			10		9	10		9
BW	5	5	5	3	3	8	9		7	9		7
V_{pv}	13			11			16			16		
V_{liver}	16		7	10		6	14		5	14		5
V_{ss}	1	1	1	2	2	2	7			8		
CL_R	15			16			13			11		
A_{CYP3A4}	4	4	4	5	5	3	3	3	2	3	3	2
A_{CYP3A5}	3	3	3	4	4	4	1	1	1	1	1	1
A_{UGT1A4}	12		6	13		5	8		4	7		4

*For the exSobol and Sobol, numbers underlined indicate input parameters with sensitivity index >0.01 and <0.1, i.e. parameters having a only moderate impact on the outputs.

+For Morris screening, all input parameters were ranked based on its GI metric.

Reference

1. Zi, Z., *Sensitivity analysis approaches applied to systems biology models.* IET Syst Biol, 2011. **5**(6): p. 336-6.
2. Sobol′, I.M., *Global sensitivity indices for nonlinear mathematical models and their Monte Carlo estimates.* Mathematics and Computers in Simulation, 2001. **55**(1): p. 271-280.
3. Gan, Y., et al., *A comprehensive evaluation of various sensitivity analysis methods: A case study with a hydrological model.* Environmental Modelling & Software, 2014. **51**(Supplement C): p. 269-285.
4. Marino, S., et al., *A methodology for performing global uncertainty and sensitivity analysis in systems biology.* J Theor Biol, 2008. **254**(1): p. 178-96.
5. Saltelli, A., S. Tarantola, and K.P.S. Chan, *A Quantitative Model-Independent Method for Global Sensitivity Analysis of Model Output.* Technometrics, 1999. **41**(1): p. 39-56.
6. Saltelli, A., et al., *Global Sensitivity Analysis. The Primer*. 2008, John Wiley & Sons, Ltd.
7. Iooss, B. and P. Lemaître, *A review on global sensitivity analysis methods*, in *Uncertainty management in Simulation-Optimization of Complex Systems: Algorithms and Applications*, C. Meloni and G. Dellino, Editors. 2015, Springer.
8. Song, X., et al., *Global sensitivity analysis in hydrological modeling: Review of concepts, methods, theoretical framework, and applications.* Journal of Hydrology, 2015. **523**(Supplement C): p. 739-757.
9. Kucherenko, S., S. Tarantola, and P. Annoni, *Estimation of global sensitivity indices for models with dependent variables.* Computer Physics Communications, 2012. **183**(4): p. 937-946.
10. Li, G., et al., *Global sensitivity analysis for systems with independent and/or correlated inputs.* J Phys Chem A, 2010. **114**(19): p. 6022-32.

11. Most, T., *Variance-based sensitivity analysis in the presence of correlated input variables.* Proceedings 5th International Conference on Reliable Engineering Computing (REC), Brno, 2012.
12. Xu, C. and G. Gertner, *Extending a global sensitivity analysis technique to models with correlated parameters.* Computational Statistics & Data Analysis, 2007. **51**(12): p. 5579-5590.
13. Xu, C. and G.Z. Gertner, *Uncertainty and sensitivity analysis for models with correlated parameters.* Reliability Engineering & System Safety, 2008. **93**(10): p. 1563-1573.
14. Zuniga, M.M., S. Kucherenko, and N. Shah, *Metamodelling with independent and dependent inputs.* Computer Physics Communications, 2013. **184**(6): p. 1570-1580.
15. Mara, T.A. and S. Tarantola, *Variance-based sensitivity indices for models with dependent inputs.* Reliability Engineering & System Safety, 2012. **107**(Supplement C): p. 115-121.
16. Vu-Bac, N., et al., *Uncertainty quantification for multiscale modeling of polymer nanocomposites with correlated parameters.* Composites Part B: Engineering, 2015. **68**(Supplement C): p. 446-464.
17. Sobol, I.M. and Y.L. Levitan, *On the use of variance reducing multipliers in Monte Carlo computations of a global sensitivity index.* Computer Physics Communications, 1999. **117**(1): p. 52-61.
18. Wentworth, M.T., R.C. Smith, and H.T. Banks, *Parameter Selection and Verification Techniques Based on Global Sensitivity Analysis Illustrated for an HIV Model.* SIAM/ASA Journal on Uncertainty Quantification, 2016. **4**(1): p. 266-297.
19. Henkel, T., H. Wilson, and W. Krug, *Global sensitivity analysis of nonlinear mathematical models - an implementation of two complementing variance-based algorithms*, in *Proceedings of the Winter Simulation Conference*. 2012, Winter Simulation Conference: Berlin, Germany. p. 1-12.
20. Morris, M.D., *Factorial Sampling Plans for Preliminary Computational Experiments.* Technometrics, 1991. **33**(2): p. 161-174.
21. McNally, K., R. Cotton, and G.D. Loizou, *A Workflow for Global Sensitivity Analysis of PBPK Models.* Front Pharmacol, 2011. **2**: p. 31.
22. Bilal, N., *Implementation of Sobol's method of global sensitivity analysis to a compressor simulation model.* 2014.
23. Sobol, I.M., *Sensitivity estimates for nonlinear mathematical models.* Mathematical Modelling and Computational Experiments, 1993. **1**(4): p. 407-414.
24. Homma, T. and A. Saltelli, *Importance measures in global sensitivity analysis of nonlinear models.* Reliability Engineering & System Safety, 1996. **52**(1): p. 1-17.
25. Saltelli, A., et al., *Variance based sensitivity analysis of model output. Design and estimator for the total sensitivity index.* Computer Physics Communications, 2010. **181**(2): p. 259-270.
